# Patterned pre-sensory spontaneous activity drives the structural refinement of developing cochlear ribbon synapses

**DOI:** 10.3389/fnsyn.2026.1730181

**Published:** 2026-03-23

**Authors:** Victoria C. Halim, Lukas Hallbrucker, Jan F. Ahrend, Cristian Setz, Roos A. Voorn, Samira Franke, Vanessa Konrad, Alina Seiler, Tina Pangršič, Stefan Roesler, Christian Vogl

**Affiliations:** 1 Auditory Neuroscience Group, Institute of Physiology, Medical University Innsbruck, Innsbruck, Austria; 2 Institute for Auditory Neuroscience and InnerEarLab, University Medical Center Goettingen, Goettingen, Germany; 3 Ca_v_X – Calcium Channels in Excitable Cells PhD Program, Medical University Innsbruck, Innsbruck, Austria; 4 Department of Otolaryngology, Head and Neck Surgery, University Medical Center Goettingen, Goettingen, Germany; 5 Collaborative Research Centre 889 ‘Cellular Mechanisms of Sensory Processing’, Goettingen, Germany; 6 Intas Science Imaging Instruments GmbH, Goettingen, Germany

**Keywords:** activity manipulation, auditory system development, optical stimulation device, optogenetics, sensory synapses, synapse maturation

## Abstract

**Introduction:**

In the mammalian cochlea, hearing relies on highly specialized ribbon-type synapses between sensory inner hair cells (IHCs) and postsynaptic spiral ganglion neurons. During early postnatal maturation, structural and functional refinements re-shape synaptic morphology and thereby maximize release efficiency in the run-up to hearing onset. This developmental period is further characterized by the occurrence of pre-sensory spontaneous activity waves, which are essential for the functional maturation of the ascending auditory pathway– yet, their importance for IHC presynaptic structural refinement remains uncertain.

**Methods:**

To investigate activity-dependent structural plasticity at cochlear ribbon synapses, we combined genetic, pharmacological, and optogenetic approaches with immunohistochemical and electrophysiological analyses. Moreover, we developed a novel optical stimulation device (OSD) that enables millisecond-precise, long-term and differentially-patterned optogenetic activation of cochlear IHCs under tightly controlled conditions within a standard tissue culture incubator.

**Results:**

Using this experimental framework, we show that positive as well as negative activity modulation triggers dynamic and rapidly-inducible homeostatic scaling of ribbon synapse morphology. Moreover, our data indicate that the temporal pattern of the presynaptic activity acts as a fundamental regulatory component of this process.

**Discussion:**

Our results suggest that – prior to hearing onset – pre-sensory synaptic activity plays a critical role in shaping cochlear ribbon synapse architecture in the developing auditory system.

## 1 Introduction

Accurate sound perception enables an organism to navigate within a complex three-dimensional space, pinpoint the location of sound emitters and perform inter-individual communication. In mammals, the transformation of incoming acoustic signals into neural code occurs in the sensory epithelium of the cochlea. Here, auditory inner hair cells (IHCs) translate sound-evoked basilar membrane motion into presynaptic neurotransmitter release events that transfer the sound information onto primary afferent auditory nerve fiber terminals. For this purpose, IHCs employ specialized ‘ribbon’ synapses – which achieve ultrafast, temporally precise and indefatigable synaptic release. Ribbons are proteinaceous electron-dense bodies whose structural backbone is mainly composed of the proteins Ribeye and Piccolino and act as the master scaffolds at the IHC active zone (AZ) (Schmitz et al., 2000; Magupalli et al., 2008; Regus-Leidig et al., 2013; Jean et al., 2018; Michanski et al., 2023). Mature ribbons are anchored at the presynaptic AZ membrane via Bassoon and cluster L-type Ca*_v_* 1.3 channels at their base. Moreover, IHC AZs apparently lack key neuronal presynaptic proteins, including the synaptic vesicle (SV) Ca^2+^-sensors synaptotagmins 1 and 2, priming factors of the Munc13 and CAPS families as well as complexins from their SV release machinery (Khimich et al., 2005; Strenzke et al., 2009; Beurg et al., 2010; Vogl et al., 2015). Instead, IHC vesicles contain otoferlin, a large multi-C2 domain single pass membrane protein that is thought to act as the main Ca^2+^-sensor for SV fusion and facilitates SV replenishment at IHC AZs (Roux et al., 2006; Pangrsic et al., 2010; Michalski et al., 2017). Postsynaptically, the main scaffolds postsynaptic density protein 95 (PSD95) and Homer1 support the stabilization of a single large ring-shaped glutamate receptor cluster that is mainly composed of GluA2-4 and NMDARs (Usami et al., 1995; Matsubara et al., 1996; Zhang-Hooks et al., 2016; Rutherford et al., 2023).

During postnatal development, it is well established that ribbon-type presynaptic AZs undergo a structural refinement process, in which immature multi-ribbon AZs are re-shaped into predominantly single ribbon-bearing synaptic contacts prior to hearing onset (Sobkowicz et al., 1982, 1986; Wong et al., 2014; Michanski et al., 2019; Voorn and Vogl, 2020). After translocation to the developing AZ – a process likely involving microtubule-based transport (Michanski et al., 2019; Hussain et al., 2024; Voorn et al., 2024) – ribbon precursors either attach or fuse at the presynaptic plasma membrane in a seemingly dynamic manner that coincides with the occurrence of spontaneous Ca^2+^-driven activity waves *in vitro* and *in vivo* (Tritsch et al., 2010; Johnson et al., 2013; Sendin et al., 2014; Babola et al., 2018; Harrus et al., 2018; De Faveri et al., 2025). This sensory-independent activation is an essential prerequisite for adequate auditory pathway maturation toward hearing onset (Clause et al., 2014) and has recently been shown to play an important role in downstream spiral ganglion neuron (SGN) subtype differentiation (Petitpré et al., 2018; Shrestha et al., 2018; Sun et al., 2018).

While the origin and cellular mechanism of propagation of these spontaneous Ca^2+^ waves are still areas of intense research, they have previously been proposed to depend on stochastic ATP release from cochlear supporting cells. This process then indirectly triggers IHC firing via a complex autocrine signaling cascade and is further fine-tuned by efferent cholinergic input from medial olivocochlear neurons (Tritsch et al., 2010; Johnson et al., 2011, 2013; Roux et al., 2011; Clause et al., 2014; Sendin et al., 2014; Wang and Bergles, 2015; Babola et al., 2020). Regardless of the exact underlying mechanism, a regulatory role of such pre-sensory activity on IHC ribbon synapse morphology and function may be suspected. In line with this hypothesis, pharmacological and genetic manipulations of presynaptic Ca^2+^ influx induced homeostatic ribbon scaling in developing lateral line neuromast hair cell ribbons of zebrafish larvae (Sheets et al., 2012). Moreover, activity-dependent structural plasticity could also be demonstrated in various other ribbon-bearing systems in mammals: For instance, illumination-dependent structural changes could be observed at rod photoreceptor synaptic ribbons in mice (Adly et al., 1999; Spiwoks-Becker et al., 2004), where photo-stimulation resulted in the attenuation of ribbon length alongside a compression of the presynaptic Ca*_v_* 1.4 and RIM2 cluster size (Dembla et al., 2020). Similarly, in rat pinealocytes, synaptic ribbons were demonstrated to undergo diurnal changes, in which ribbons increase in size during darkness, while stimulus deprivation of the pineal gland due to constant light exposure resulted in ribbon-shrinkage (Spiwoks-Becker et al., 2008). Such structural adaptations occurred alongside differential recruitment of a range of cytomatrix proteins, including Piccolo and Bassoon. Yet, while a clear picture regarding activity-dependent ribbon plasticity emerges in other sensory systems, the molecular mechanisms and consequences of such processes on the early assembly and developmental maturation of mammalian auditory ribbon-type AZs are still largely elusive – especially regarding rapid adaptive changes upon alterations in cellular homeostasis.

Hence, we set out to address this issue by employing a comprehensive experimental approach to assess the effects of perturbed synaptic function on ribbon morphology in developing IHCs. In addition to conventional approaches for spontaneous activity modulation – such as pharmacological or genetic manipulation, which provide rather global insights into AZ structural plasticity during postnatal maturation – we also assessed the effects of more subtle alterations in the spontaneous firing rates. For this latter purpose, we developed a novel optogenetics-based methodology to achieve vastly improved spatiotemporal resolution via highly customizable photo-depolarization of IHCs within the excised organ of Corti kept in short-term organotypic culture. This way, we could interrogate the effects of distinct temporal depolarization patterns with exquisite cell-type specificity. Intriguingly, a similar approach has previously been used to demonstrate that the extent and position of the axon initial segment (AIS) – a highly specialized neuronal structure involved in action potential initiation and propagation – is regulated by the level and temporal patterning of incoming stimuli, thus enabling input-specific and highly dynamic fine-tuning of neuronal excitability (Grubb and Burrone, 2010). To now investigate if such processes also instruct AZ morphology and thereby regulate release site efficiency in the developing cochlea via ribbon size regulation, we set out to first establish if IHC ribbon synapse size is indeed modulated in an activity-dependent manner using pharmacological and genetic manipulations. Subsequently, we employed patterned optogenetic stimulation to investigate the effects of differential temporal presentation of the incoming stimuli in more detail. For this latter purpose, we conceived an optical stimulation device (OSD) that allows for fully customizable and long-term optical stimulation of organ of Corti explant cultures within the tightly-controlled environment of a standard tissue culture incubator.

In summary, we present evidence that auditory ribbon synapses are strikingly dynamic scaffolds that can rapidly adapt to altered activation patterns via homeostatic scaling of the pre- and especially the postsynapse. In addition, we provide a detailed description of our custom-built OSD with the aim of disseminating a valuable community tool for other researchers investigating synaptic plasticity or other dynamic, activity-dependent cellular processes.

## 2 Materials and methods

### 2.1 Animals

Studies were conducted either on C57BL/6 J mice (The Jackson Laboratory, Stock No: 000664), *Otof*-KO mice (Reisinger et al., 2011), *Ca_v_1.3*-KO mice (Platzer et al., 2000), or an optogenetic mouse line that was generated by crossbreeding transgenic knock-in (KI) mice expressing Cre recombinase under the IHC-specific vesicular glutamate transporter (*Vglut3*) promoter (Jung et al., 2015) with conditional knock-in mice carrying a ChR2(H134R)-eYFP fusion gene with a loxP-flanked STOP cassette (Ai32; The Jackson Laboratory, Stock No: 024109) (Madisen et al., 2012). Transcription of the downstream ChR2(H134R)-eYFP fusion gene will only occur when the STOP cassette is deleted in Cre-expressing tissues – in this case resulting in IHC-specific ChR2-eYFP expression (Ai32-Vglut3-Cre-KI; Chakrabarti et al., 2022). All employed ChR2-expressing animals were *Ai32 fl/fl^cre+/cre-^*.

All experiments were conducted according to national, regional and institutional guidelines of either the University Medical Center Göttingen (Lower Saxony/Germany), or Innsbruck Medical University (Tyrol/Austria). The herein used mildly burdened *Otof*- and *Ca_v_1.3*-KO mouse lines were bred with adequate breeding licenses (AZ19/3134 in Göttingen and AZ2024-0.796.090 in Innsbruck, respectively).

All mouse line details, reagents and technical resources employed in this manuscript can be found in Supplementary Table S1.

### 2.2 Organotypic cultures

Organ of Corti explant cultures were isolated from 5-day-old (P5) C57BL/6 J or Ai32-Vglut3-Cre mice. In a subset of our experiments, we used both, Cre positive (Ai32cre+) and Cre negative (Ai32cre-) littermates as additional specificity controls. After decapitation, heads of the pups were kept in a 15 mL Falcon tube containing dissection buffer, composed of HBSS (1X) (Gibco, 14,025–092), 10 mM HEPES (Gibco, 15,630–049), 250 ng/mL fungizone (Life Technologies, 15,290–026), and 10 μg/mL penicillin G (Sigma, P3032-25MU). Each head was then hemisected along the sagittal suture and, following removal of the brain, the cochleae dissected from the temporal bone. The cochlear bone and the stria vascularis were peeled off, revealing the three turns of the spiraling basilar membrane, which supports the organ of Corti. After the extreme apical and basal turns of the cochlear spiral were clipped off, Reissner’s and the tectorial membrane were carefully removed, leaving the apico-medial turn of the organ of Corti with exposed rows of IHCs and outer hair cells (OHCs). Finally, upon final excision, organ of Corti explants were either mounted on a Cell-Tak (Corning, 354240)-coated coverslip, 35 mm diameter (MatTek Corporation, P35G-1.5-14-C) or 50 mm diameter (FluoroDish, FD5040) glass-bottom dish filled with growth medium [Neurobasal-A medium (1X) (Gibco, 12349-015) with 1% GlutaMAX (100X) (Gibco, 35050-061), 1% B27 (50X) (Life Technologies, 17504044), and 1.5 μg/mL ampicillin (Sigma, A0166)]. All experiments were conducted on P5 inner ear tissue that was subsequently kept in organotypic culture either for one day (DIV1; all immunohistological analyses after pharmacological or optogenetic treatments) or one to two days (DIV1-2) for electrophysiological experiments.

### 2.3 Acute preparation of organ of Corti explants

Acute organ of Corti explants were isolated from 6-day-old (P6) C57BL/6 J or C57BL/6NJ mice. Following decapitation, heads were placed in phosphate-buffered saline (PBS, 1X, Sigma-Aldrich, P4417) and hemisected along the sagittal suture. After removal of the brain, cochleae were removed from the temporal bone and following the aforementioned dissection procedure, organs of Corti were fixed for 1 h in ice-cold 4% formaldehyde (FA; diluted in PBS). Acute preparations of P6 littermates were then compared with organotypic cultures at P5DIV1 to investigate the degree of preservation regarding synaptic innervation and general structural integrity of the tissue.

### 2.4 Short-term high-speed time-lapse calcium imaging

Short-term time-lapse imaging experiments were performed at an Abberior Instruments Expert Line STED microscope, operated in confocal laser scanning mode, using a 60x/1.20 NA water immersion objective. A top mount on-stage incubator (Okolab uno stage top incubator, H391-Olympus-IX-SUSP 2015) was used to create and maintain environmentally-controlled conditions (37 °C, 5% CO_2_). Pharmacological treatments were performed by drop-wise bulk application of 100 μL culture medium in between time-lapse imaging sessions. Isradipine (Sigma Aldrich, I6658) was applied to a final concentration of 10 μM; BayK8644 (Tocris #1544), to a final concentration of 5 μM. Time-lapse images were acquired over a 10-min period, and consisted of single-plane optical sections, at intervals of 0.78 s. Region of interest (ROI) selection was based on GCaMP6 signal strength. Spatial dimensions were kept consistent at 70×35 μm. GCaMP6 fluorescence was acquired in confocal mode at 5 μs dwell time with a pinhole size of 3.6 AU.

### 2.5 Image processing of short-term high-speed time-lapse imaging

The fluorescent GCaMP6 signal was processed in FIJI/ImageJ, by extraction of the fluorescence intensity (F) over time. To create a ROI consisting solely of the SGN fluorescence, an image mask was generated based on the maximum intensity projection, followed by intensity thresholding and noise reduction. The extracted fluorescence intensity was then baseline corrected (F_GCaMP6_ = ΔF-F_0_). For quantification of the GCaMP6 peak frequency, the FIJI/ImageJ BAR plugin was used (https://zenodo.org/records/28838).

### 2.6 Electrophysiological recordings

For direct comparability, whole-cell recordings of organotypically-cultured IHCs were performed in growth medium at room temperature (RT) using the perforated patch-clamp method as described in previous work (Vogl et al., 2015). In brief, remnants of the tectorial membrane, OHCs, pillar cells and inner phalangeal cells were mechanically removed using glass micropipettes of varying sizes via a micromanipulator (Sutter Instruments, MP-285). Patch pipettes were pulled from borosilicate glass capillaries (Science Products GmbH, GB150-8P) with a P-97 flaming/brown micropipette puller (Sutter Instruments). Patch pipette tips were dipped in amphotericin-free intracellular solution (ICS) and backfilled afterwards with ICS containing (in mM): 150 KCl, 10 HEPES, 1 MgCl_2_ and 250 μg/mL amphotericin B (EMD Millipore Corp., USA). The pH was adjusted to 7.2 with KOH and the osmolarity was ~289 mmol/kg. Recordings were carried out using an EPC-10 amplifier (HEKA Elektronik) controlled by Patchmaster software. Recordings were started once the series resistance was below 30 MΩ and all voltages were corrected for the liquid junction potential (+1.74 mV; calculated using LJPcalc at T: 22 °C) during offline analysis. Spontaneous activity was recorded in current clamp configuration in absence of current injection (*I*inj: 0 pA). Cells with membrane leak current over −50 pA at the standard holding potential of −70 mV in voltage clamp mode were discarded. IHC photo-stimulation was achieved using a blue LED (center wavelength at λ = 475 nm; X-cite Turbo, Excelitas). Irradiance and duration of the light pulses were controlled using an EPC-9 amplifier. A FITC filter set was used to transmit the LED light onto the culture preparation. Radiant flux (mW) was measured with a photodiode-based power meter (Thorlabs) that was placed under the 40x objective lens used for patching. The diameter of the illumination spot was measured using a green fluorescent slide and stage micrometer, then used for power density calculations (mW/mm^2^). Photo-depolarizations were recorded in current-clamp mode.

### 2.7 Light-stimulation experiments using the optical stimulation device (OSD)

Long-term optogenetic stimulations were implemented using a custom-built OSD, which was constructed of two independent – yet, synchronously activated – illumination units (i.e., stimulation and non-stimulation control), each employing one blue LUXEON Z LED array (SZ-05-H3; LUXEON) covering a 2.6 mm^2^ emitter area nested on top of an aluminum heat sink. These illumination units were housed inside a waterproof metal box, which was enclosed by a piece of 3 mm thick milk glass plate (transmission: 78%) that acted as a light diffuser to homogenize the illumination spot at the sample plane above the LED array. On top of the glass was a three-dimensionally (3D) printed plastic receiver plate with one LED-exposed and one LED-covered area, on which the stimulated and contralateral non-stimulated (control) culture dishes were placed, respectively. Optical stimulation was either presented as 5 × 5 ms pulse bursts applied at a frequency of 50 Hz (one burst per second) followed by a rest period prior to the next stimulation episode (“*oBurst*”), or 5 × 5 ms continuous stimulation (“*oSparse*”) that was evenly spaced throughout the experiment (total light pulse count per condition: 18,000). Unless stated otherwise, all stimulation paradigms were applied for 1 h in growth medium in a standard incubator at 37 °C with 5% CO_2_ to mimic physiological conditions as closely as possible. To control for excessive and unphysiological tissue heating, the sample temperature was constantly monitored using an external temperature probe attached to the sidewall of the stimulated dish. Experiments would have automatically been terminated in case the sample temperature reached 40 °C – however, this did not occur a single time with the final OSD version. All temperature measurements regarding specimen heating analysis were performed in a medium-filled culture dish at the sample plane to mimic the real-life condition as closely as possible.

### 2.8 Pharmacological treatments

Organ of Corti explant cultures were exposed to the Ca^2+^ channel inhibitor isradipine, the Ca^2+^ channel agonist (±)-BayK8644 – both dissolved in dimethylsulfoxside (DMSO) (Sigma, 2650) and diluted to the final concentrations (i.e., isradipine: 10 μM; BayK8644: 5 μM) in growth medium directly prior to the application – for the indicated durations at 37 °C/5% CO_2_. Since isradipine is light sensitive, all steps have been performed under minimum light conditions. In parallel, DMSO was used as vehicle control to treat the contralateral ear of the same animal for each treatment condition. Cultures were immediately fixed for immunohistochemistry after the end of each treatment.

### 2.9 Immunohistochemistry, confocal and STED microscopy

Organotypic cultures and acute preparations were fixed with 4% FA for 1 h on ice. After fixation, samples were permeabilized with 0.5% Triton-X100 in PBS for 30 min, followed by blocking with a PBS-based blocking buffer containing 0.5% Triton-X100 and 10% goat serum for 1 h at RT. Afterwards, specimens were incubated with primary antibodies (2 h at RT) and, after extensive washing, with adequate secondary reagents (Supplementary Table S1) prior to mounting in fluorescence mounting medium (ProLong™ Glass Antifade Mountant, Life Technologies Corporation, P36984). Image acquisition was performed on an Olympus IX83-based Abberior Instruments Expert Line 2-color STED microscope (Abberior Instruments GmbH) in confocal and/or STED mode using a 1.4 NA UPlanSApo 100x oil immersion objective. We employed 561 nm and 640 nm laser lines for excitation and a pulsed 775 nm laser for stimulated emission depletion. Image stacks were acquired with Imspector Software with pixel sizes of 80 × 80 nm and step sizes of 200 nm (confocal). Pixel sizes in 2D-STED equated to 15 × 15 nm in xy.

### 2.10 Chemical stimulation and cFos immunohistochemistry

Organotypic cultures were incubated in resting solution (in mM): NaCl 136.5, CaCl_2_ 1.3, KCl 5, MgCl 1, HEPES 10, or stimulating solution (in mM): NaCl 95, CaCl_2_ 5, KCl 40, MgCl 1, HEPES 10 for 2 h, or exposed to optical stimulation for 1 h at 37 °C with 5% CO_2_. Cultures were then fixed for 1 h with 4% FA and stained with anti-cFos, anti-Tuj1, and either TO-PRO3™-3 Iodide or anti-GFP for cellular context (Supplementary Table S1).

### 2.11 Image processing and analysis

The analysis of Ca*_v_* 1.3 and CtBP2 area from 2D-STED images was carried out using a custom-written routine in ImageJ (version 1.54f). Individual channels were processed by applying a Gaussian blur low-pass filter (sigma = 1), followed by masking after maximum entropy dark thresholding. Touching objects were separated with the watershed algorithm, and objects larger than 50 pixels were fitted as ellipsoids, with their areas measured, respectively, for Ca*_v_* 1.3 and CtBP2. Correlation between Ca*_v_* 1.3 and CtBP2 areas was assessed using a linear regression in Prism 10.4.1 (GraphPad Software, LLC).

Quantitative analyses of synaptic ribbon counts, integrated fluorescence intensities and volumes were performed using the Imaris software package (Bitplane, Zürich, Switzerland). For all conditions except *Ca_v_1.3*-KO, datasets were analyzed in Imaris x64 9.6.1, with 3D reconstructions of ribbon puncta generated using the surface algorithm with local background subtraction enabled. Reconstruction parameters were set to a surface detail of 0.048 μm and a maximum sphere diameter of 0.280 μm. Touching ribbon puncta were separated at 0.150 μm with a quality filter applied. PSDs were rendered with a surface detail of 0.150 μm and a maximum sphere diameter of 0.520 μm. For *Ca_v_1.3-*KO samples, analysis was conducted in Imaris x64 10.2.0. Reconstruction parameters for ribbons were adjusted to a surface detail of 0.140 μm and a maximum sphere diameter of 0.250 μm. Touching ribbon puncta were separated at 0.350 μm with a quality filter applied. PSDs were reconstructed using a surface detail of 0.163 μm and a maximum sphere diameter of 0.611 μm. In all conditions, ribbon synaptic engagement was analyzed by classifying ribbons according to their distance to the closest PSD. To be considered as synaptically-engaged, the shortest Imaris-generated surface-to-surface distance was required to be <0.5 μm. All ribbon counts were normalized to the number of IHCs in the ROI. Only cells that were fully covered in the acquired volume were rendered and analyzed.

### 2.12 Statistical analysis

All datasets were statistically analyzed using Prism 9.1.2 apart from the STED imaging data, for which we used a later version (10.4.1) (GraphPad Software, LLC). Normality was tested with Shapiro–Wilk test, while comparisons between grouped data sets were done as appropriate with either Dunn’s multiple comparisons test, Dunnett’s T3 multiple comparisons test, Mann–Whitney U- test, or Welch’s t-test, as mentioned at the respective position in the text. *N* indicates number of animals, while *n* indicates number of data points. Means, indicated by *M*, are given with ± SEM, medians are indicated by *Mdn*, and interquartile ranges by *IQR*. The statistical significance for each of the comparisons is indicated by the symbols *n.s.* for not significant (*p* > 0.05), *p < 0.05, **p < 0.01, and ***p < 0.001, respectively. All relevant source data are deposited on Mendeley Data for public access (Mendeley Data, doi: 10.17632/fkxph7grn5.1).

## 3 Results

### 3.1 Ribbon size determines the Cav1.3 clustering capacity of a given synaptic contact

**Figure 1:**
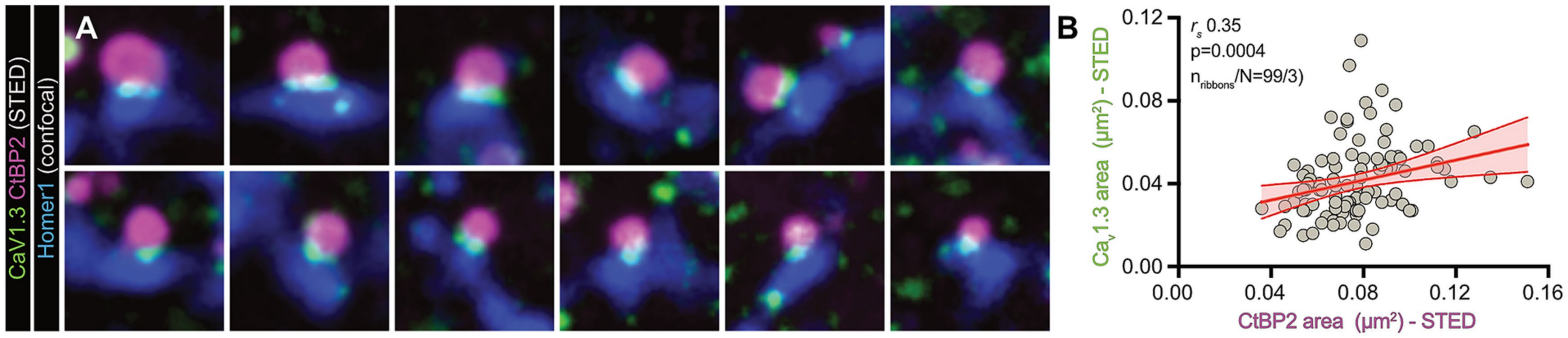
2D-STED nanoscopy reveals a positive correlation between the sizes of ribbons and Ca*_v_*1.3 clusters. **(A)** Representative examples of single optical sections of IHC synapses stained with antibodies against Ca*_v_*1.3 (green; KO-verified) and CtBP2 (magenta) visualized using 2-color 2D-STED nanoscopy, while Homer1 (blue) – indicating the PSD context – was acquired in confocal mode. The individual image dimensions are 1 × 1 μm. **(B)** Presynaptic ribbon and Ca*_v_*1.3 cluster sizes obtained from 2D-STED images display a moderate positive correlation as assessed by CtBP2 and Ca*_v_*1.3 areas. The red line indicates a linear regression with the associated 95% confidence interval. *N*_animals_ = 3, *n*_ribbons_ = 99; *r*_s_ = 0.35, ***p = 0.0004.

To date, multiple lines of evidence suggest a major AZ scaffolding role of the synaptic ribbon, which determines various structural and functional parameters of a given afferent contact. For example, ribbon size and SV packing density increase in synchrony during postnatal maturation (Michanski et al., 2019). Moreover, in murine post-hearing IHCs, chick basilar papilla hair cells as well as zebrafish retinal rod bipolar cells, ribbon size positively correlates with presynaptic Ca^2+^ influx as well as Ca*_v_*1.3 cluster size (Martinez-Dunst et al., 1997; Frank et al., 2009, 2010; Ohn et al., 2016; Karagulyan and Moser, 2023; Rameshkumar et al., 2025). Hence, ribbon size serves as a morphological proxy for SV release capacity of a given AZ. To now establish if similar observations can already be made during early postnatal development, where functional Ca*_v_*1.3 channels are still widely distributed in the basolateral membrane (Wong et al., 2014), we employed super-resolution 2D-STED nanoscopy to analyze the relationship between ribbon size and the associated Ca^2+^ channel complement at the IHC AZ membrane of acutely dissected P5 organs of Corti using a commonly-employed and KO-validated antibody against Ca*_v_*1.3 (Figure 1). We found a moderate positive correlation (Figures 1A,B) between ribbon size and Ca*_v_*1.3 cluster area, which indicates that ribbon size indeed dictates presynaptic Ca^2+^ channel cluster size at developing AZs, albeit to a lesser degree than after synapse maturation has concluded (Karagulyan and Moser, 2023). Hence, dynamic regulation of ribbon size – for example via pre-sensory spontaneous activity during postnatal maturation – could provide a means for functional adaptation and fine-tuning of afferent signaling.

### 3.2 Short-term pharmacological activity modulation triggers homeostatic scaling of developing auditory ribbon synapses

**Figure 2:**
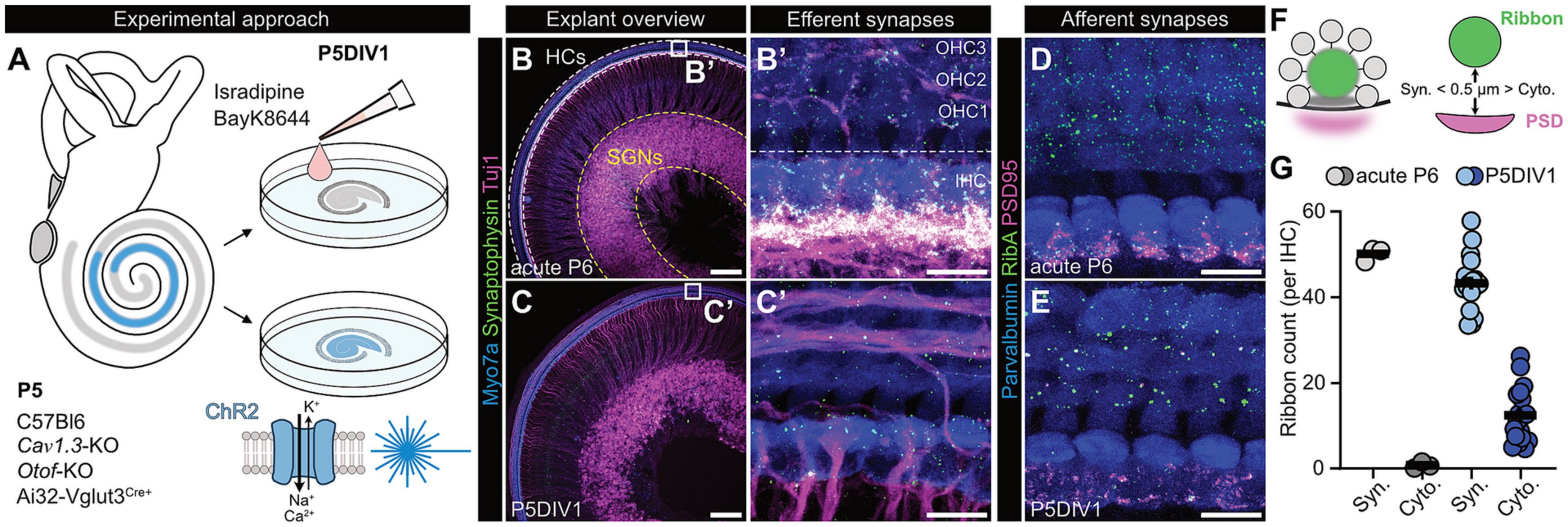
Experimental overview of the short-term organotypic culture-based model system. **(A)** Cochleae were harvested from mouse pups at postnatal day P5. A segment of the apico-medial turn of the organ of Corti was dissected for organotypic explant cultures (indicated in blue). Explant cultures were then either acutely treated with pharmacological modulators or exposed to optical stimulation after one day *in vitro* (P5DIV1). **(B,C)** Representative confocal maximum z-projections of **(B)** a whole-mount preparation at P6 and **(C)** a P5DIV1 organotypic culture from the apico-medial turn of the organ of Corti. Hair cells (HCs) are stained against Myosin 7a (blue), efferent olivocochlear terminals against Synaptophysin (green), and afferent spiral ganglion neurons (SGN) against Tuj1 (magenta). The HC region is outlined with a white dashed line, the SGN somata encircled with a yellow dashed line for orientation. Scale bars: 100 μm. **(B’,C’)** Representative confocal maximum z-projection of the HC regions from panel **(B,C)**. The border between single row IHCs and three rows of OHCs (OHC1-3) is indicated by the dashed line. Scale bars: 10 μm. **(D,E)** Representative maximum projections of confocal z-stacks in the HC region of **(D)** an acute preparation at P6 and **(E)** an organotypic explant culture at P5DIV1 to illustrate afferent synapses. HCs are stained against Parvalbumin (blue), the presynaptic ribbon marker RibeyeA (green), and the postsynaptic density marker PSD95 (magenta). Scale bars: 10 μm. **(F)** Schematic drawing to illustrate the distance criterium for ribbon classification: To be considered as synaptically-engaged, the fluorescence-based shortest Imaris-generated surface-to-surface distance was required to be <0.5 μm. Cyto., cytoplasmically-floating ribbons; Syn., synaptic ribbons. **(G)** Average ribbon counts per IHC in acutely dissected organs of Corti at P6 (N_animals_ = 3, n_Corti_ = 3) versus a pooled set of the investigated control ribbon counts from the subsequent Figures 3, 4 and 7 (i.e., isradipine, *Otof*-KO, BayK, oSparse, oBurst) at P5DIV1 (N_animals_ = 17, n_Corti_ = 17).

This initial finding further motivated us to investigate the underlying mechanisms of ribbon size regulation and structural plasticity – and in particular the effects of presynaptic activity on these processes. As mentioned above, developing IHCs are characterized by stimulus-independent, Ca^2+^-based action potential firing, which is critical for adequate cochlear development (Beutner and Moser, 2001; Johnson et al., 2011, 2013; Sendin et al., 2014; Harrus et al., 2018; Shrestha et al., 2018; Sun et al., 2018). Based on this knowledge and inspired by previous work on zebrafish that employed pharmacological and genetic manipulations to investigate activity-dependent ribbon plasticity in lateral line neuromast hair cells (Sheets et al., 2012), we first investigated the impact of presynaptic silencing or overactivation on the architecture of IHC ribbon-type AZs that were kept in short-term organotypic culture (Figure 2). Such cultures maintain near native structural integrity and importantly, retain a major fraction of their afferent innervation, thus serving as a valuable model system for in-depth ribbon synapse analysis (Figures 2A–D). Yet, it is worth noting that we observed a non-negligible dissection-and/or culture-related reduction in afferent synapse counts when compared to age-matched acute preparations (on average amounting to ~14% in the herein employed culture system) and in addition, major loss of modulatory efferent olivocochlear fiber input. The latter was evident from the strongly reduced Tuj1 fluorescence below the IHC basal poles and the associated stark reduction in synaptophysin labeling that specifically labels efferent terminals in age-matched acute preparations, both clear indicators of significant efferent fiber loss (Figures 2B–G).

**Figure 3:**
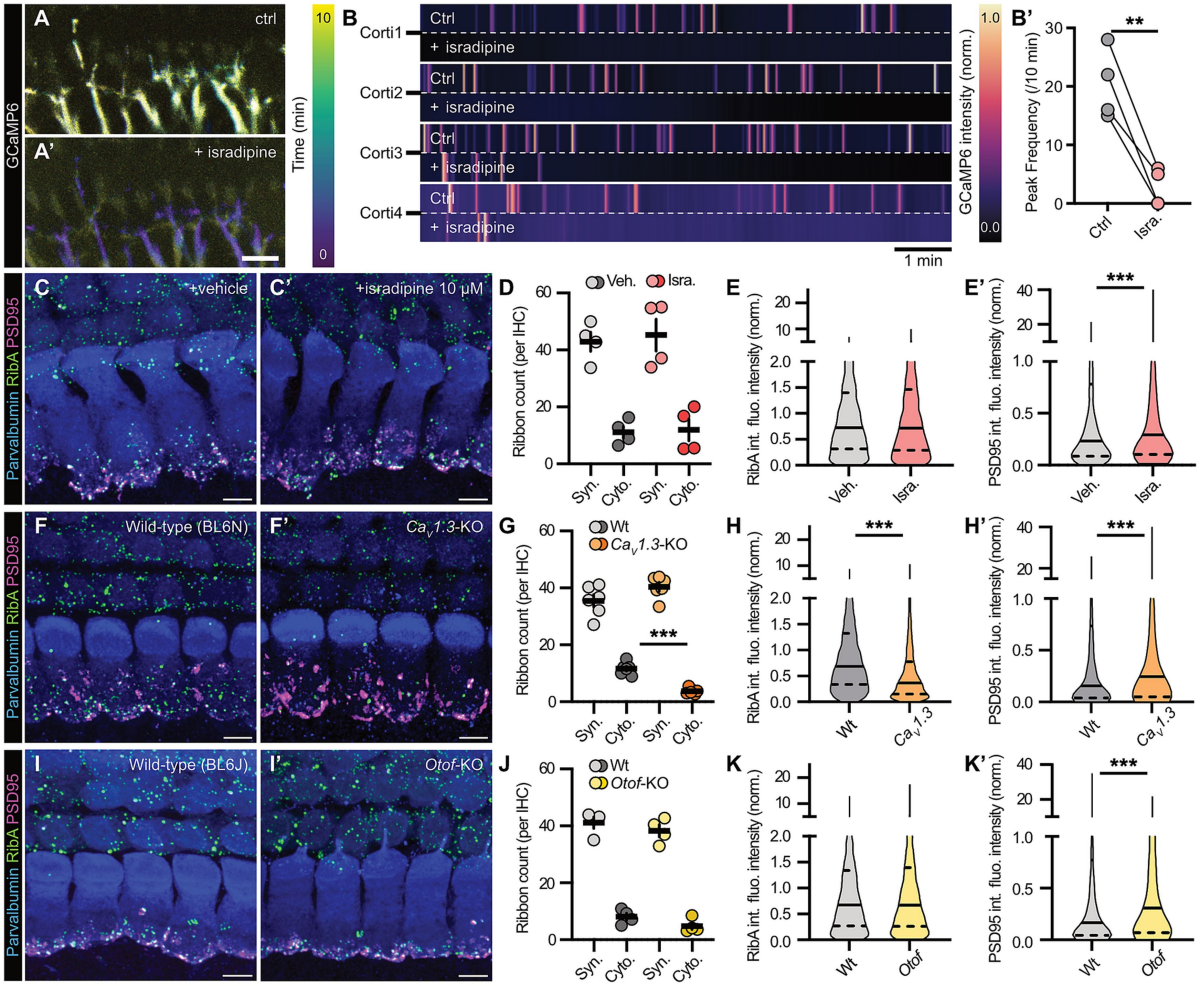
Effects of acute pharmacological inhibition and genetics-based functional disruption of murine auditory ribbon synapses. **(A)** Representative temporal maximum intensity projections of GCaMP6 fluorescence in afferent SGN terminals over a 10 min timelapse interval in an organotypically-cultured organ of Corti of early postnatal Snap25-T2A-GCaMP6s-D mice (P5DIV1). Pharmacological block of presynaptic Ca*_v_*1.x-mediated Ca^2+^ influx by application of isradipine (Isra., 10 μM) abolished the spontaneous synaptic activity detected in the postsynaptic SGNs; panels display a representative time interval prior **(A)** and post **(A’)** isradipine application. Here, across the analysis time window, early Ca^2+^ waves are represented in dark blue, late activity waves in yellow – the latter are completely absent upon isradipine treatment, thus illustrating complete block of synaptic transmission in this condition upon wash-in of the drug. Scale bar: 10 μm. **(B)** Normalized GCaMP6 fluorescence intensity plots of four different organ of Corti cultures treated with isradipine, prior and post application. **(B’)** Quantification of the peak frequency in SGN terminal GCaMP6 fluorescence, showing a dramatic reduction of activity bursts upon isradipine application. (*N*_animals_ = 4, *n*_Corti_ = 4, imaged prior and post application). Paired Student’s *t*-test: **p < 0.01. **(C,C’)** Representative maximum projections of confocal *z*-stacks of WT organ of Corti cultures treated with either **(C)** vehicle (DMSO) or **(C’)** 10 μM isradipine for 1 h at P5DIV1. IHCs were stained against Parvalbumin (blue), RibeyeA (green) and PSD95 (magenta). Scale bars: 5 μm. **(D)** Average ribbon counts in vehicle- and isradipine-treated IHCs at P5DIV1 (*N*_animals_ = 4, *n*_Corti_ = 4 for both conditions) reveal no statistically significant differences between treatments. Two-way ANOVA with Holm-Šídák’s multiple comparisons correction: n.s. = no significant. **(E,E’)** Violin plots showing **(E)** unchanged integrated fluorescence intensity of presynaptic ribbons, but **(E’)** an increase in PSD integrated fluorescence intensity in isradipine-treated IHCs at P5DIV1 after 1 h of treatment. *N*_animals_ = 4, *n*_Corti_ = 4, *n*_ribbons_ = 2,524, *n*_PSD_ = 1,038 for vehicle- and *N*_animals_ = 4, *n*_Corti_ = 4, *n*_ribbons_ = 2,713, *n*_PSD_ = 1,076 for isradipine-treated conditions for both conditions. Median is indicated by solid lines inside the violin plots, while upper and lower quartiles are indicated by dashed lines. Mann–Whitney *U*-test: ***p < 0.001. **(F,F’)** Representative maximum projections of confocal *z*-stacks of **(F)** WT and **(F’)** *Ca_v_1.3*-KO organ of Corti cultures at P5DIV1. IHCs were stained against Parvalbumin (blue), RibeyeA (green) and PSD95 (magenta). Scale bars: 5 μm. **(G)** Quantification of average ribbon counts reveals a decrease in cytosolic ribbons in *Ca_v_1.3*-KO IHCs at P5DIV1 (*N*_animals_ = 3, *n*_Corti_ = 6 for both genotypes). Two-way ANOVA with Holm-Šídák’s multiple comparisons correction: ***p < 0.001. **(H,H’)** Violin plots reveal **(H)** a decrease in ribbon integrated fluorescence intensity and **(H’)** an increase in PSD integrated fluorescence intensity in *Ca_v_1.3*-KO IHCs at P5DIV1. *N*_animals_ = 3, *n*_Corti_ = 6, *n*_ribbons_ = 2,555, *n*_PSD_ = 1,575 for each WT and *N*_animals_ = 3, *n*_Corti_ = 6, *n*_ribbons_ = 2,555, *n*_PSD_ = 1,466 for *Ca_v_1.3*-KOs. Median is indicated by solid lines inside the violin plots, while upper and lower quartiles are indicated by dashed lines. Mann–Whitney *U*-test: ***p < 0.001. **(I,I’)** Representative maximum projections of confocal *z*-stacks of **(I)** WT and **(I’)** *Otof*-KO organ of Corti cultures at P5DIV1. IHCs were stained against Parvalbumin (blue), RibeyeA (green) and PSD95 (magenta). Scale bars: 5 μm. **(J)** Quantification of synaptic as well as cytoplasmic ribbon counts reveals WT-like ribbon numbers in *Otof*-KO IHCs at P5DIV1 (*N*_animals_ = 3, *n*_Corti_ = 6 for both genotypes). Two-way ANOVA with Holm-Šídák’s multiple comparisons correction. **(K,K’)** Violin plots showing **(K)** unchanged integrated fluorescence intensity in ribbons but **(K’)** an increase in PSD integrated fluorescence intensity in *Otof*-KO IHCs at P5DIV1. *N*_animals_ = 3, *n*_Corti_ = 6, *n*_ribbons_ = 3,912, *n*_PSD_ = 1,423 for each WT and *N*_animals_ = 3, *n*_Corti_ = 6, *n*_ribbons_ = 3,105, *n*_PSD_ = 1,064 *Otof*-KO. Median is indicated by solid lines inside the violin plots, while upper and lower quartiles are indicated by dashed lines. Mann–Whitney *U*-test: ***p < 0.001. Refer to Supplementary Figure S1 for ribbon/PSD volume data.

To first analyze the effects of activity suppression on afferent synapse morphology, we employed three distinct model systems: (i) acute pharmacological blanket inhibition of L-type Ca*_v_*1.x channels using the commonly-used dihydropyridine antagonist isradipine (10 μM; Figures 3A–E’), (ii) genetic blockade of presynaptic Ca^2+^ influx in a constitutive *Ca_v_1.3*-KO mouse model (Platzer et al., 2000) (Figures 3F–H’) and (iii) selective presynaptic disruption of SV fusion using a global *Otof*-KO mouse line (Reisinger et al., 2011) (Figures 3I–K’). While *Ca_v_1.3*-KO IHCs completely fail to initiate excitation-secretion coupling, Ca*_v_*1.3-mediated Ca^2+^ influx remains intact in *Otof*-KO IHCs – yet, synaptic exocytosis is virtually abolished in this latter line (Roux et al., 2006; Reisinger et al., 2011; Vogl et al., 2015). Thus, these two mouse lines offer excellent model systems to study the effects of different aspects of synaptic inhibition on auditory ribbon synapse morphology. On the other hand, pharmacological inhibition via isradipine application serves as an acute manipulation control that circumvents developmental compensation issues that might arise in the mutant mouse lines. To analyze the synaptic architecture, we then labeled synaptic complexes immunohistochemically with antibodies against RibeyeA and PSD95 to visualize presynaptic ribbons and mark juxtaposed postsynaptic scaffolds, respectively. From the acquired image stacks, we extracted ribbon counts, integrated fluorescence intensities and volumes of synaptically-engaged ribbons as well as their associated PSD95-positive postsynaptic patches (Figure 3; Supplementary Figure S1). Consistent with previous findings (Sheets et al., 2012), our data indicate that cochlear IHC ribbon morphology is surprisingly resistant to functional silencing during the advanced stages of maturational refinement – regardless of the experimental system. However, as an exception to this rule, we observed a compaction of ribbon size in *Ca_v_1.3*-KO IHCs (Figures 3F–H’), which might arise from impaired formation due to cumulative lack of Ca^2+^-dependent molecular motor activity – as has been observed for example for deafness-related Myosin 6 (Batters et al., 2016) – and hence insufficient supply of synaptic components during postnatal maturation – as indicated by the reduction in cytoplasmically-floating ribbon precursors we observed in this mouse line (Figure 3G). However, if this hypothesis holds true will have to be tested in future experiments. Interestingly, across all inhibitory conditions, the associated PSDs appeared to be prone to homeostatic upscaling to compensate presynaptic loss-of-function, a finding in line with previous reports on mouse mutants with impaired presynaptic glutamate release (Kim et al., 2019; Stalmann et al., 2021; Karagulyan and Moser, 2023; Oestreicher et al., 2024).

**Figure 4:**
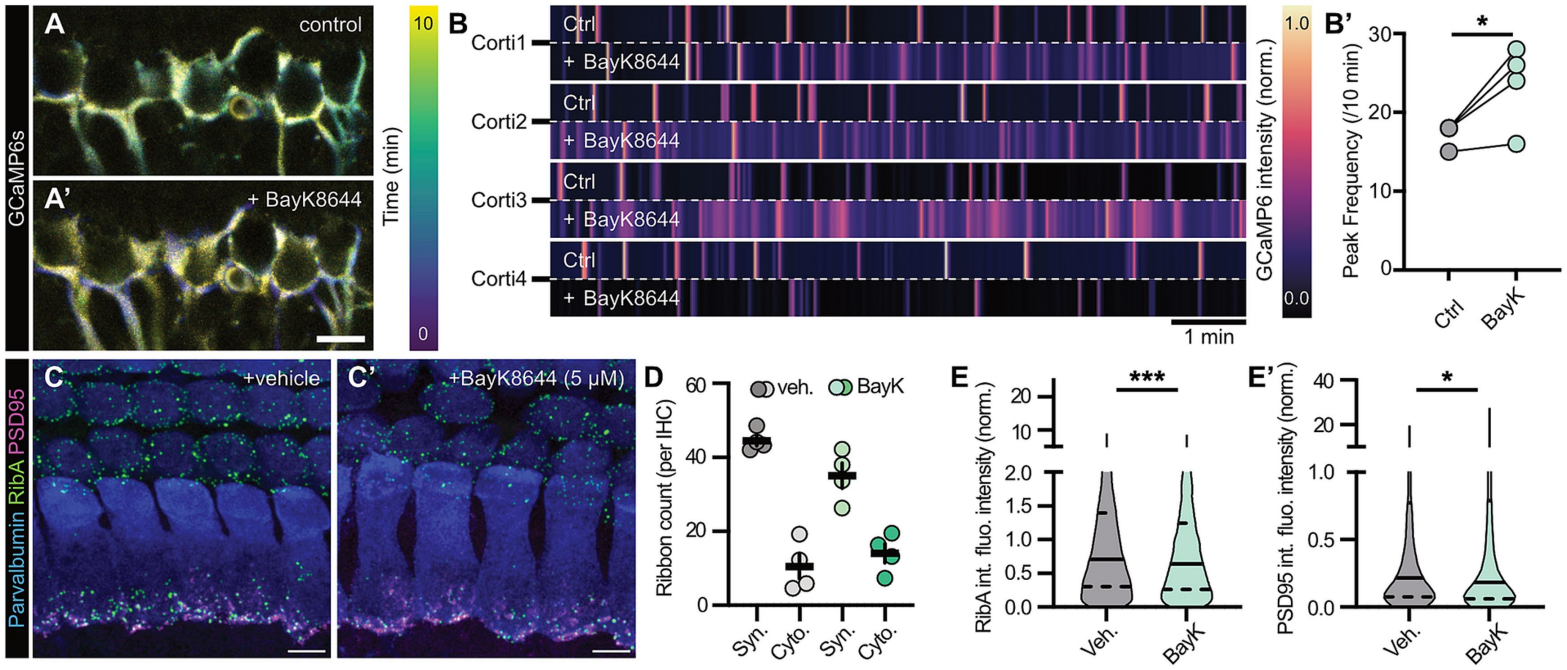
Effects of acute pharmacological activation of murine auditory ribbon synapses. **(A)** Representative temporal maximum projections of GCaMP6 intensity in afferent SGNs over a 10 min timelapse interval in organ of Corti cultures of early postnatal Snap25-2A-GCaMP6s-D mice (P7DIV1), prior **(A)** and post **(A’)** pharmacological treatment with Ca*_v_*1.x agonist BayK8644 (BayK, 5 μM). Across the analysis time window, early Ca^2+^ waves are represented in dark blue, late activity waves in yellow. Scale bar: 10 μm. **(B)** Normalized GCaMP6 fluorescence intensity plots of four different organ of Corti cultures treated with BayK, prior and post application. **(B’)** Quantification of the peak frequency in SGN GCaMP6 fluorescence, showing increased postsynaptic activity upon BayK application. (*N*_animals_ = 4, *n*_Corti_ = 4, imaged prior and post application). Paired *t*-test: *p < 0.05. **(C, C’)** Representative maximum projections of confocal *z*-stacks of WT organ of Corti cultures treated with either **(C)** vehicle (DMSO) or **(C’)** 5 μM BayK for 1 h at P5DIV1. IHCs were stained against Parvalbumin (blue), RibeyeA (green) and PSD95 (magenta). Scale bars: 5 μm. **(D)** Quantification of synaptic as well as cytoplasmic ribbon counts of vehicle- or BayK-treated IHCs at P5DIV1 reveals normal ribbon numbers regardless of the pharmacological manipulation (*N*_animals_ = 4, *n*_Corti_ = 4 for control and BayK) after 1 h of treatment. Two-way ANOVA with Holm-Šídák’s multiple comparisons correction. **(E,E’)** Violin plots reveal **(E)** a mild decrease in both ribbon and **(E’)** PSD integrated fluorescence intensities in BayK-treated IHCs at P5DIV1. *N*_animals_ = 4, *n*_Corti_ = 4, *n*_ribbons_ = 2,565, *n*_PSD_ = 1,153 for vehicle- and *N*_animals_ = 4, *n*_Corti_ = 4, *n*_ribbons_ = 2078, *n*_PSD_ = 1,022 for BayK-treated conditions after 1 h of treatment. Median is indicated by solid lines inside the violin plots, while upper and lower quartiles are indicated by dashed lines. Mann–Whitney *U*-test: *p < 0.05, ***p < 0.001. Refer to Supplementary Figure S1 for ribbon/PSD volume data.

Our next experiments aimed to functionally reverse the stimulation paradigms and trigger increased presynaptic activity – again inspired by previous work on zebrafish that used the L-type Ca*_v_*1.x channel activator BayK8644 (BayK; 5 μM) for this purpose (Sheets et al., 2012) (Figure 4). In our system, BayK application clearly elevated the postsynaptic Ca^2+^ spike frequency in the afferent terminals of organotypic cultures from Snap25-T2A-GCaMP6s mice and seemingly also elevated the basal Ca^2+^ levels within the auditory nerve terminals, both findings indicative of globally increased activity levels and challenged intracellular Ca^2+^ buffering (Figures 4A–B’). Analogous to the zebrafish work, we found that artificially increased activity levels led to compensatory homeostatic downscaling of the synaptic complex, this time involving both, the presynaptic ribbon as well as the associated PSD, thus indicating that positive modulation acts as a stronger trigger for structural plasticity at maturing ribbon synapses (Figures 4C–E’).

While we focused our analyses on integrated fluorescence intensities for better comparability with previous studies (Sheets et al., 2012) of ribbons and PSDs, we additionally assessed ribbon and PSD95 volumes, the observed global trends were largely maintained, but less obvious – in particular for the acute pharmacological manipulations (Supplementary Figure S1). This finding might hence indicate alterations in the clustering density of the investigated molecular components, such as molecular enrichment versus structural dispersion within the pre- and postsynaptic scaffolds, that precede physical scaling of the synaptic complex and is likely owed to the relatively brief duration of the pharmacological treatments (1 h). To test this hypothesis, we attempted long-term treatments with isradipine and BayK (12 h), which however were not tolerated well by IHCs and SGNs, and led to structural degeneration that prevented meaningful analysis at this point (data not shown).

### 3.3 Establishing an optogenetic model system to investigate the effects of differential temporal spike patterning on ribbon synapse structure

We next focused on the role of temporal patterning of depolarization events. This approach was motivated by previous reports from CNS neurons that showed that distinct activity patterns can prompt drastic adaptive responses to rapidly modulate the size and position of the AIS and thus fine-tune cellular excitability (Grubb and Burrone, 2010). Interestingly, in the developing auditory system, it has been suggested that the spontaneous IHC firing patterns differ along the tonotopic axis, with apical IHCs displaying burst firing, while basal IHCs show rather continuous spiking (Johnson et al., 2011; but see Sendin et al., 2014). Therefore, we asked if alterations in the stimulus presentation of spontaneous firing might play a role in the functional maturation of the peripheral auditory pathway – for example, by inducing adaptive plasticity of ribbon synapse morphology.

**Figure 5:**
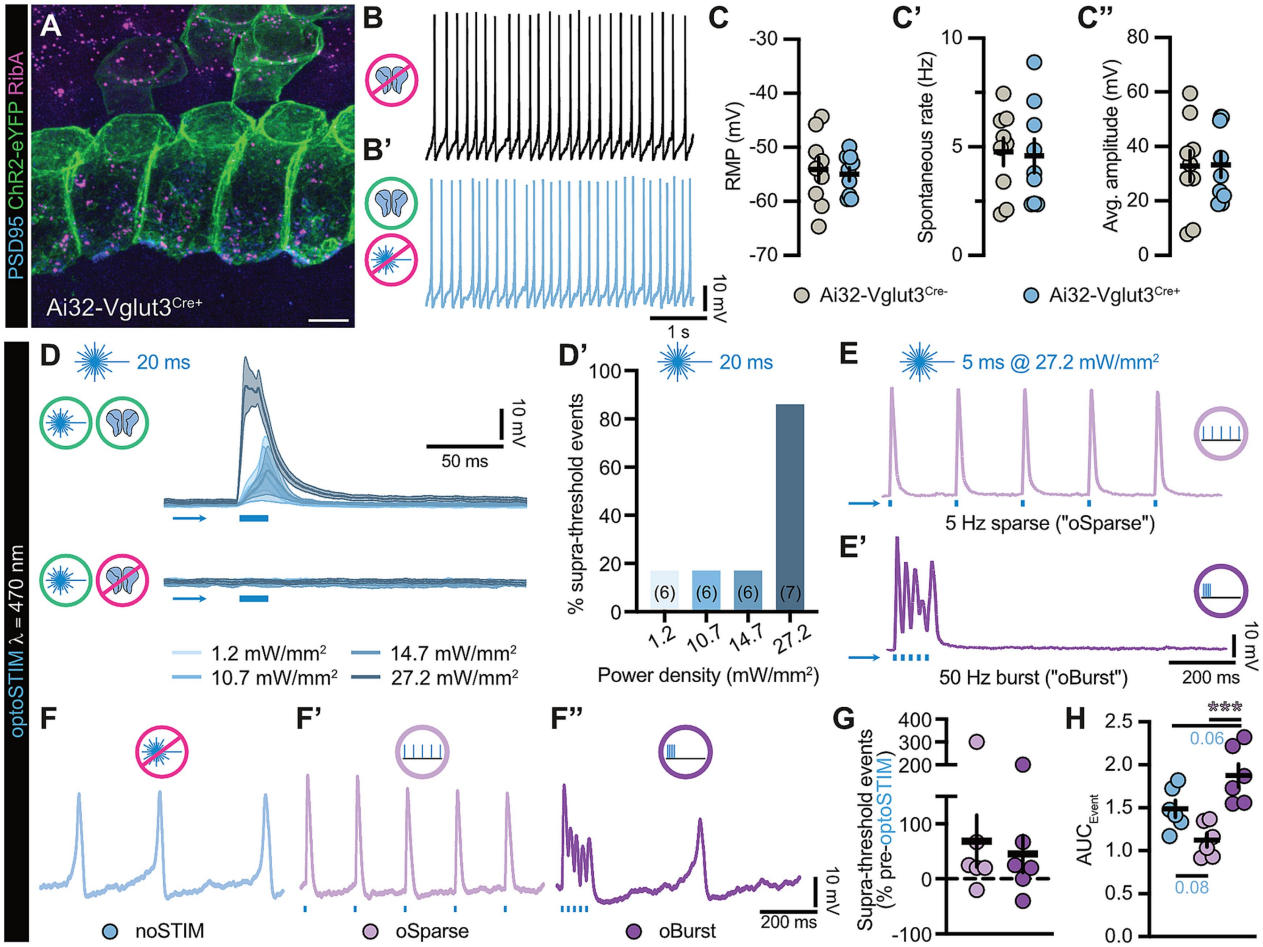
Toward acute and differentially patterned optogenetic activity modulation of murine auditory IHCs. **(A)** ChR2-expression in the IHC plasma membrane of an Ai32-Vglut3^Cre+^ mouse. IHCs were immunostained for PSD95 (blue), ChR2-eYFP (green) and RibeyeA (magenta). Scale bar: 5 μm. **(B,B’)** Representative spontaneous activity recordings of IHCs from an Ai32-Vglut3^Cre-^ mouse and an Ai32-Vglut3^Cre+^ littermate at I_inj_ = 0 pA. **(C–C”)** Quantification of **(C)** resting membrane potential (RMP), **(C’)** spontaneous rate, and **(C”)** average amplitude in Ai32-Vglut3^Cre+^. *N*_animals_ = 7, *n*_IHC_ = 9 for Ai32-Vglut3^Cre-^ and *N*_animals_ = 7, *n*_IHC_ = 9 for Ai32-Vglut3^Cre+^. Mann–Whitney *U*-test: not significant. **(D)** Example traces of photo-depolarization amplitudes in P5DIV1–2 Ai32-Vglut3^Cre+^ IHCs evoked by 20 ms of blue light stimulation with 1.2, 10.7, 14.7, and 27.2 mW/mm^2^ laser power density at *λ* = 470 nm from a hyperpolarized membrane potential of −71.74 mV to suppress spontaneous spiking. **(D’)** Quantification of supra-threshold events (here, light-evoked responses) given in percentage of optical stimulation at indicated PDs. AP threshold was set to V_m_: −35 mV (Sendin et al., 2014). (*n*) values are shown in each column within the figure. **(E,E’)** Example traces of **(E)** 5 Hz optically-triggered and evenly spaced (“*oSparse*”) or **(E’)** 50 Hz burst (“*oBurst*”) optical stimulation protocols, each – in total – lasting 1 s. Each protocol consisted of five 5 ms light pulses delivered at an intensity of 27.2 mW/mm^2^. For the *oSparse* protocol, light pulses were triggered every 200 ms, whereas for the *oBurst* protocol, pulses occurred every 20 ms upon hyperpolarizing current injection to suppress spontaneous firing. **(F–F”)** Example traces of IHC activity from the respective RMP (I_inj_ = 0 pA) recorded for 1 s under either of the three conditions: **(F)** No optical stimulation (noSTIM), **(F’)** 5 Hz *oSparse,* or **(F”)** 50 Hz *oBurst*. **(G)** Quantification of supra-threshold events reveal an increased firing rate during *oSparse* and *oBurst* optical stimulation protocols. *N*_animals_ = 3, *n*_IHC_ = 6. *oSparse*: 68.6 ± 47.6%; *oBurst*: 45.3 ± 34.0% above spontaneous rate. **(H)** Quantification of the area under the curve (AUC) for spontaneous vs. optically-triggered supra-threshold events. *N*_animals_ = 3, *n*_IHC_ = 6. noSTIM: 1.49 ± 0.09; *oSparse*: 1.13 ± 0.08; *oBurst*: 1.88 ± 0.13. One-way ANOVA with Šídák’s multiple comparisons correction: ***p = 0.0005 for *oSparse* vs. *oBurst*.

While pharmacological manipulation suffers from poor temporal resolution and lacks cell-type selectivity, genetic disruption is prone to developmental compensation effects. We hence decided to employ optogenetics for these experiments and selectively expressed algal channelrhodopsin-2 H134R (ChR2) in the IHC plasma membrane (Figure 5) using an *Ai32-Vglut3-Cre* mouse line, which shows highly consistent and homogenous expression of the tagged optogene in the basolateral IHC membrane (Chakrabarti et al., 2022) (Figure 5A). Upon basic electrophysiological characterization, unstimulated ChR2-positive IHCs exhibit seemingly normal functional properties that were consistent with previous reports (Beutner and Moser, 2001; Johnson et al., 2011, 2013) (Figures 5B–C”) – albeit with the added benefit of being reliably activatable to fire Ca^2+^ spikes upon exposure to short blue light flashes (Figures 5D,D’). Inspired by previous work (Grubb and Burrone, 2010; Glebov et al., 2017), we then tested two distinct stimulation paradigms to assess potential effects of differentially presented stimulus patterns: (i) a continuous and evenly spaced 5 Hz sparse optical stimulation protocol (“*oSparse*”) – and (ii) a grouped 50 Hz burst firing protocol (“*oBurst*”) that rapidly triggered a transient but large bolus Ca^2+^ influx event (Figures 5E,E’), though with temporal control and IHC specificity. To our surprise, when applying these stimulation paradigms to IHCs at their native resting membrane potential, both protocols failed in drastically altering the spontaneous firing rate, but – in particular regarding the *oSparse* paradigm – appeared to synchronize and lock the intrinsic firing rate to our imposed stimulation pattern. Interestingly, spike integral analysis revealed that the optically-triggered *oSparse* triggered spikes showed a strong trend toward lower area under the curve (AUC) values due to the almost instantaneous nature of optically-induced depolarization versus the slow pacemaker-like ramping toward firing threshold that was observed for naturally occurring Ca^2+^ spikes (Figures 5F–H). In contrast, *oBurst* firing events displayed drastically increased AUCs (due to superposition of events) and were at times additionally characterized by intrinsically generated intermediate spikes during the interstimulus interval periods.

### 3.4 Design and validation of a novel custom-built in-incubator optical stimulation device for faithful long-term activity modulation under tightly controlled conditions

**Figure 6:**
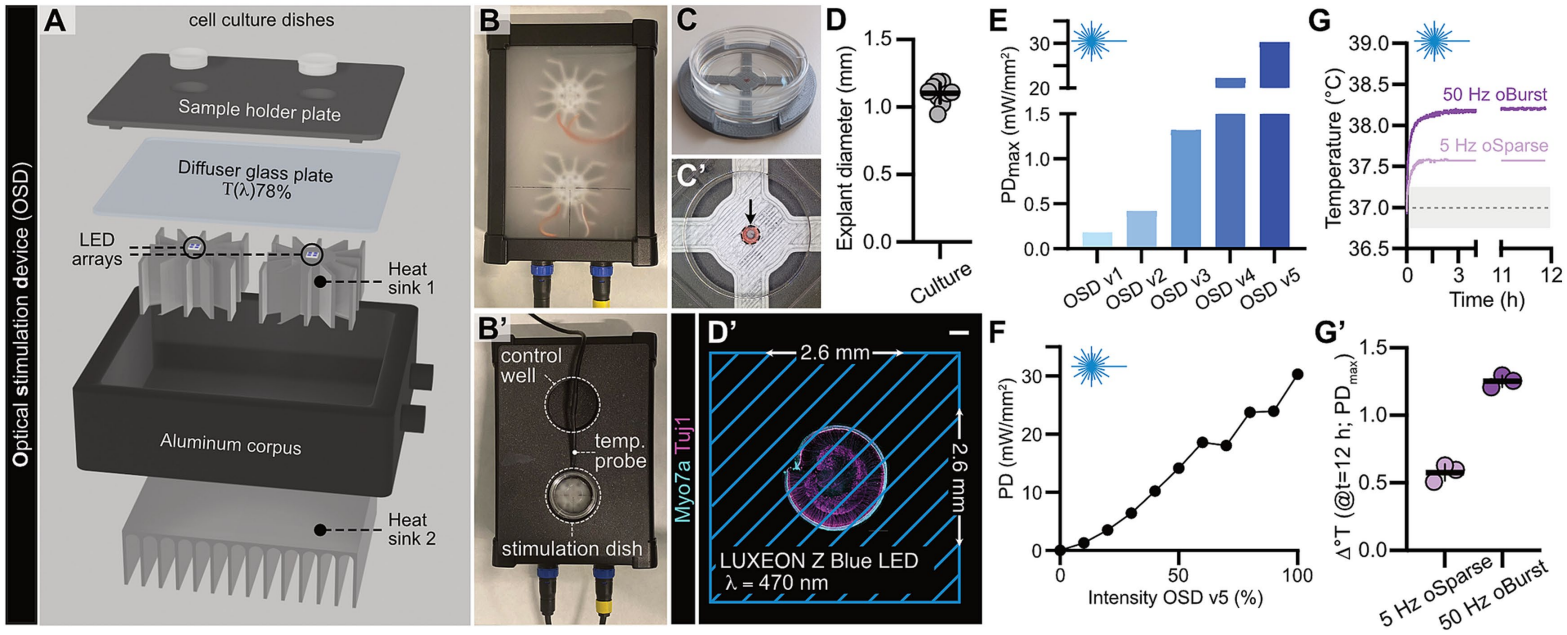
Design concept and functional characterization of a custom-built in-incubator optical stimulation device (OSD) to interrogate stimulation-dependent synapse plasticity in cochlear IHCs. **(A)** Schematic representation of the OSD (generated in Blender 4.5.4 software). The device consists of a water-tight aluminum corpus that houses two LED arrays mounted on aluminum heat sinks for thermal regulation. A diffuser glass plate with a transmittance [T(λ)] of 78% is placed above the LED arrays to evenly distribute light across the sample area. A 3D-printed sample holder plate is positioned at the top to accommodate cell culture dishes and secure them in the correct position above the LED arrays. **(B,B’)** Top view of the operational OSD **(B)** without the sample holder plate and **(B’)** with the sample holder plate in place, highlighting the key functional components: a control well for non-stimulated samples, a temperature probe for monitoring thermal conditions, and a designated stimulation dish area for optical stimulation experiments. **(C,C’)** Photograph of a cell culture dish placed on a custom-designed and 3D-printed mounting guide, which facilitates the precise positioning of organ of Corti explant cultures for optical stimulation experiments and thus ensures optimal positioning in respect to the LED light sources. **(C’)** A magnified view of the mounting area on the guide is shown, with the arrowhead indicating a centrally-mounted organ of Corti explant culture. **(D)** Scatter dot plot showing the measured diameters of organ of Corti explant cultures, with an average diameter of 1.10 ± 0.02 mm. **(D’)** Schematic representation of the illumination area generated by LUXEON Z blue LED (λ = 470 nm). The illuminated region measures 2.6 mm x 2.6 mm (based on LED array perimeter) and is depicted with blue diagonal lines. A representative organ of Corti explant culture is comfortably positioned within the illumination field with the organ of Corti stained against Myosin7a (cyan) and Tuj1 (magenta). Scale bar: 200 μm. **(E)** Bar graphs illustrating the maximum measured power density (PD) in the sample plane achieved by various iterations of the OSD (for detailed information on the individual LED configurations and power outputs across v1–5, please refer to the Supplementary Figure S2). **(F)** LED power measurements of the final OSD version (v5) demonstrate a maximum power output of ~30.0 mW/mm^2^ at 100% intensity. **(G,G’)** Representative real-life temperature measurements were conducted in the sample plane using either *oBurst* or *oSparse* protocols to determine tissue heating during ongoing optical stimulation. **(G’)** The maximum temperature change for each optical stimulation protocol was calculated at the end of the stimulation cycle and plotted as scatter dot plots, *N* = 3.

While these experiments provided us with information on adequate light doses to ensure faithful control of IHC excitability and validate our different stimulation paradigms, it is (i) an extremely low throughput task and (ii) the tissue perturbance produced by sealing a comparably massive glass electrode onto a single mechanically-cleaned IHC prevents long-term optogenetic manipulation in an otherwise near-native functional state with intact afferent innervation. Therefore, we conceived a novel in-incubator optical stimulation device (OSD) to be able to specifically activate intact ChR2-expressing IHCs *in situ* with the two above-tested stimulation paradigms over prolonged periods of time (Figures 6A–B’). In this context, it is important to point out that the required power density values for faithful spike triggering of ~30 mW/mm^2^ is a surprisingly high value, especially considering that the ED_50_ of the herein used ChR2/H134R construct was previously reported to be ~1.07 mW/mm^2^ on dissociated single cells (Lin, 2011). Yet, other studies that either employed the same mouse line for optogenetic analysis of IHC synaptic physiology in post-hearing mice (Chakrabarti et al., 2022) or stimulated distinct neuronal populations with conceptually similar LED-based stimulation systems reported comparably high power density values to faithfully trigger ChR2 activation (Boyden et al., 2005; Barral et al., 2019; Kobayashi et al., 2024). While the reason for this issue remains to be determined, it poses a major challenge for efficient light delivery and especially temperature management in an enclosed and humidified environment that already operates at physiological temperature. We thus took an iterative approach involving several prototype designs to resolve these issues (Supplementary Figure S2) and ultimately conceptualized an OSD with near optimal heat management and sufficient power density yield (1.3 ± 0.03 °C maximum temperature increase over a 12 h continuous *oBurst* stimulation protocol at 100% LED power output; Figures 6C–G’). To be able to exactly and reproducibly center the sensory epithelia in the glass bottom dishes and ensure exact culture mounting above the LED arrays, we utilized Blender 4.5.4 freeware (https://www.blender.org/) and a standard 3D filament printer to generate customizable culture mounting aids and sample holder plates (all corresponding files are available from the corresponding author) (Figures 6C–D’).

### 3.5 Evaluation of downstream SGN activation and adverse effects of optogenetic stimulation using common markers of immediate early gene expression and IHC apoptosis

**Figure 7:**
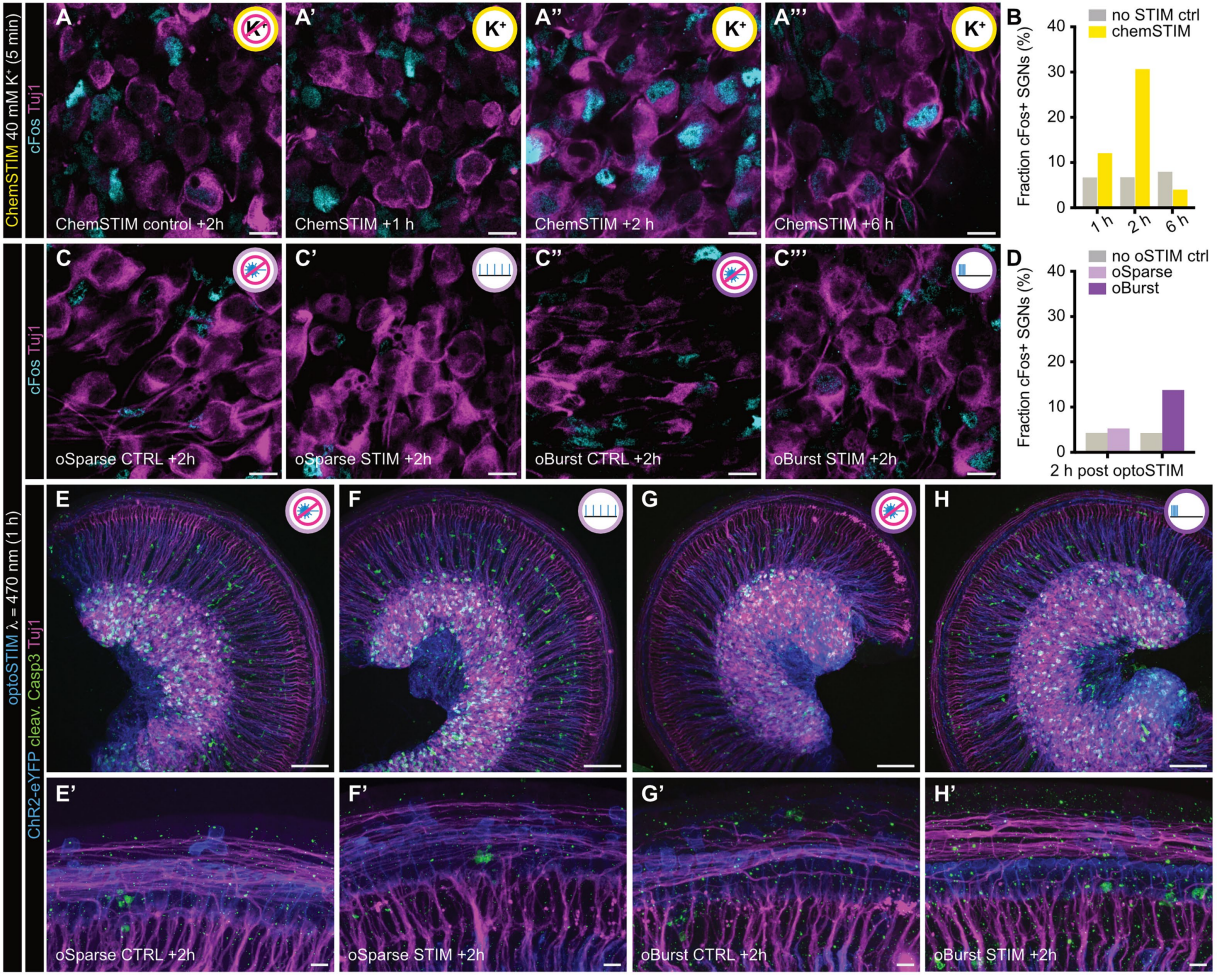
Evaluation of downstream SGN activation and potential adverse effects of optogenetic stimulation using common markers of immediate early gene expression and HC apoptosis. **(A–A”’)** Temporal expression profile of *Fos* in SGNs after 5 min application of high K^+^ (ChemSTIM). Representative confocal images of SGNs in WT organs of Corti **(A)** 2 h after application of control solution without high K^+^ concentration (ChemSTIM control), or the following recovery intervals to allow for *Fos* expression **(A’)** 1 h after ChemSTIM, **(A”)** 2 h after ChemSTIM, and **(A”’)** 6 h after ChemSTIM. SGNs were stained for cFos (cyan) and Tuj1 (magenta). Scale bars: 10 μm. **(B)** Quantification of cFos+ SGNs reveals 2 h as the optimal post-stimulation time point to detect stimulation-dependent *Fos* expression in SGNs. 1 h: *N*_animals_ = 3, *n*_Corti_ = 3 for both control and chemSTIM, 2 h: *N*_animals_ = 3, *n*_Corti_ = 3 for both control and chemSTIM, and 6 h: *N*_animals_ = 3, *n*_Corti_ = 3 for both control and chemSTIM. **(C–C”’)** cFos expression after 1 h optical stimulation (optoSTIM) of Ai32-Vglut3^Cre+^ IHCs evaluated 2 h after stimulus onset. Representative confocal images of the spiral ganglion of P5DIV1 organs of Corti 2 h after **(C)** no optical stimulation, conducted in parallel with **(C’)** *oSparse* stimulation (*N*_animals_ = 4, *n*_Corti_ = 4 for both control and *oSparse*), and **(C”)** no optical stimulation, conducted in parallel with **(C”’)** *oBurst* stimulation (*N*_animals_ = 5, *n*_Corti_ = 5 for both control and *oBurst*). SGNs were stained against cFos (cyan) and Tuj1 (magenta). Scale bars: 10 μm. **(D)** Quantification of cFos+ SGN nuclei reveals an increased occurrence after *oBurst*, but not *oSparse* optoSTIM. **(E–H)** Representative maximum *z*-projection confocal overview images of Ai32-Vglut3^Cre+^ organs of Corti 2 h after **(E)** no optical stimulation, conducted in parallel with **(F)** *oSparse* stimulation (*N*_animals_ = 3, *n*_Corti_ = 3 for both control and *oSparse*), and **(G)** no optical stimulation, conducted in parallel with **(H)** *oBurst* stimulation (*N*_animals_ = 3, *n*_Corti_ = 3 for both control and *oBurst*). Organs of Corti were stained with ChR2-eYFP (blue), Tuj1 (magenta), and cleaved Caspase-3 (green) as a marker for cell apoptosis. Scale bars: 100 μm. **(E’–H’)** High magnification maximum projections of the hair cell region of all conditions depicted in **(E–H)** illustrating that optoSTIM paradigms do not trigger excessive IHC apoptosis. Scale bars: 10 μm. Refer to Supplementary Figure S3 for additional control experiments regarding treatment specificity and cFos expression in IHCs.

Once we had established that the final OSD design operates within physiological acceptable parameters and is capable of producing sufficient irradiation levels in the culture plane, we set out to further validate OSD-based activation of our explant culture regarding two important aspects: (i) the quality of downstream activation of SGNs and (ii) potential adverse effects due to phototoxicity, which might compromise our analyses (Figure 7).

To identify active neuronal populations, immediate early gene (IEG) expression such as *Fos* is commonly used as a cell biological readout. However, recent work in hippocampal neurons revealed a cell-type specific and frequency-dependent mechanism that was not only triggered by excessively high, but also recurrent yet low spike rates, whereas intermediate firing rates failed to trigger *Fos* expression (Anisimova et al., 2023). Hence, the relationship between spiking behavior and *Fos* expression appears more complex than previously anticipated and may rather represent an inducible molecular marker that indicates departure from cellular homeostasis. We hence set out to determine if our prolonged OSD-based IHC activation paradigms would push the postsynaptic SGNs out of their homeostatic range or would rather produce stimuli within a physiological range. For this purpose, we first employed a commonly used chemical stimulation approach (chemSTIM) with a brief pulse application of high K^+^ (5 min; 40 mM K^+^) to establish if SGNs, which are known to support excessively high firing frequencies of multiple hundreds of Hz upon maturation, can at all be driven to express *Fos* during the postnatal maturation phase and, if so, determine its expression time course upon activation (Figures 7A–B). In these experiments, chemSTIM-dependent cFos could indeed be detected in ~30% of the sampled SGNs and a large number of glial cells within the spiral ganglion, with expression peaking at 2 h post stimulation and declining thereafter – thus indicating that an unnaturally strong stimulatory input can indeed initiate IEG expression in this system. We then used the same approach on our OSD-activated cultures and tested both previously established stimulation paradigms (*oSparse* and *oBurst* optoSTIM, applied for 1 h; Figures 7C–D). While *oSparse* apparently failed to trigger *Fos* expression, *oBurst* led to a moderate increase of <20% in cFos+ SGN nuclei, suggesting that recurrent burst induction on IHC level challenges downstream homeostasis in only a subpopulation of SGNs. Yet, these data also indicate that optoSTIM-based manipulation of intrinsic activation with the current settings largely operates within the physiological range. In recent years, various studies reported that recurrent blue light irradiation by itself is able to modulate gene expression – including *Fos* – in neurons and microglia (Cheng et al., 2016; Tyssowski and Gray, 2019), we therefore tested the effects of our *oBurst* blue light stimulation protocol on cFos expression in *Ai32-Vglut3^Cre-^* explant cultures, which do not express ChR2. In these additional control experiments, we failed to detect any light-dependent alterations in cFos expression (Supplementary Figures S3A,B), thus suggesting that the observed effects are indeed specific activity-dependent phenomena. Finally, in contrast to the spiral ganglion, closer inspection of the hair cell region revealed that even the *oBurst* stimulation paradigm failed to trigger any detectable cFos expression in IHCs (Supplementary Figures S3C,C’), thus indicating that IHCs cannot be driven out of their homeostatic range with the herein employed opto-STIM protocols.

Next, we focused on adverse effects that might be caused by unphysiological temperature increases or phototoxic damage. While our above-reported temperature control measurements do not suggest excessive tissue heating above physiologically-relevant values in the sample plane, detrimental phototoxic effects are more difficult to identify. To address this, we performed immunostainings against cleaved caspase-3, a common marker of apoptosis induction and compared light-exposed with control cultures, in which the light path was blocked through the design of the sample holder plate. In these experiments, no signs of excessive IHC death – at least in response to the employed 1 h illumination cycle with either optoSTIM protocol – could be detected (Figures 7E–H’; Supplementary Figures S3D–D”). In fact, independent of the treatment, hair cells generally survived the organotypic culture preparation very robustly, with no quantifiable losses. In contrast, we observed cleaved caspase3 + cells within the spiral ganglion, which likely reflects delayed SGN and/or glial cell apoptosis induction resulting from the tissue extraction procedure, as they did not appear to differ between light-exposed and non-exposed cultures.

### 3.6 Targeted long-term optogenetic stimulation differentially regulates synapse size in ChR2-expressing murine auditory IHCs

**Figure 8:**
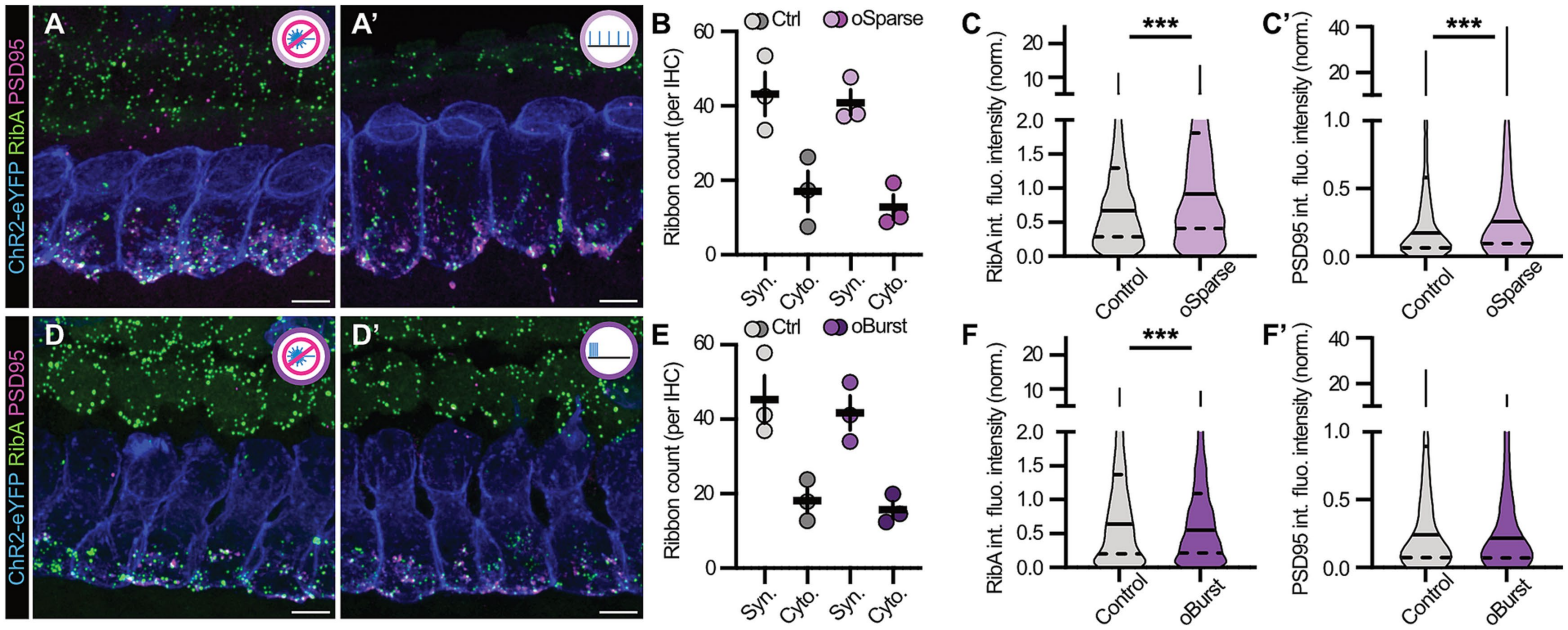
Persistent optogenetic stimulation differentially regulates synapse size in ChR2-expressing murine auditory IHCs. **(A,A’)** Representative maximum projections of confocal *z*-stacks of Ai32-Vglut3^Cre+^ IHCs exposed to either **(A)** no optical stimulation (control) or **(A’)** *oSparse* for 1 h at P5DIV1. IHCs were stained against ChR2-eYFP (blue), RibeyeA (green) and PSD95 (magenta). Scale bars: 5 μm. **(B)** Average ribbon count appears unchanged in *oSparse*-exposed IHCs at P5DIV1 after 1 h of treatment (*N*_animals_ = 3, *n*_Corti_ = 3 for control and *oSparse* stimulation). Two-way ANOVA with Holm-Šídák’s multiple comparisons correction. **(C–C’)** Violin plots reveal a statistically significant increase in both, **(C)** ribbon and **(C’)** PSD95 integrated fluorescence intensities in *oSparse*-exposed IHCs at P5DIV1 after 1 h of treatment. *N*_animals_ = 3, *n*_Corti_ = 3, *n*_ribbons_ = 1943, *n*_PSD_ = 948 for control and *N*_animals_ = 3, *n*_Corti_ = 3, *n*_ribbons_ = 1840, *n*_PSD_ = 780 for *oSparse* stimulation. Median is indicated by solid lines inside the violin plots, while upper and lower quartiles are indicated by dashed lines. Mann–Whitney *U*-test: ***p < 0.001. **(D–D’)** Representative maximum projections of confocal *z*-stacks of Ai32-Vglut3^Cre+^ IHCs exposed to either **(D)** no optical stimulation (control) or **(D’)** *oBurst* for 1 h at P5DIV1. IHCs were stained against ChR2-eYFP (blue), RibeyeA (green) and PSD95 (magenta). Scale bars: 5 μm. **(E)** Average ribbon count is unchanged in *oBurst*-exposed IHCs at P5DIV1 after 1 h of treatment (*N*_animals_ = 3, *n*_Corti_ = 3 for control and *oBurst* stimulation). Two-way ANOVA with Holm-Šídák’s multiple comparisons correction. **(F,F’)** Violin plots reveal a significant decrease in **(F)** ribbon integrated fluorescence intensities in *oBurst*-exposed IHCs at P5DIV1 after 1 h of treatment, while **(F’)** PSDs showed a similar trend that however failed to reach our criteria for statistical significance. *N*_animals_ = 3, *n*_Corti_ = 3, *n*_ribbons_ = 2038, *n*_PSD_ = 679 for control and *N*_animals_ = 3, *n*_Corti_ = 3, *n*_ribbons_ = 1875, *n*_PSD_ = 618 *oBurst*. Median is indicated by solid lines inside the violin plots, while upper and lower quartiles are indicated by dashed lines. Mann–Whitney *U*-test: ***p < 0.001. Refer to Supplementary Figure S1 for ribbon/PSD volume data.

Having convinced ourselves that we can reliably, efficiently and safely trigger optogenetic spikes with our OSD platform, we finally set out to investigate the effects of differentially-patterned optoSTIM paradigms on IHC ribbon synapse morphology (Figure 8). To our surprise, while ribbon counts appeared unaffected in both conditions (Figures 8A–B, D), *oSparse* and *oBurst* induced seemingly opposite effects: while *oSparse* led to a striking upscaling of presynaptic ribbons as well as their associated PSDs (Figures 8C,C’), *oBurst* triggered presynaptic downscaling (Figures 8F,F’). This latter observation is conceptually comparable to (yet not quite as effective as) BayK application, as it did not induce structural adaptation of the associated PSDs. This observation may hence either indicate (i) different amounts of Ca^2+^ increase due to BayK or *oBurst* application or (ii) the involvement of postsynaptic L-type Ca*_v_*1.x channels (i.e., Ca*_v_*1.2 and Ca*_v_*1.3) that likely reside in the afferent PSDs, but would not be affected by purely presynaptic activity modulation, as is the case with the *oBurst* scenario. In contrast, due to the lack of cell type specificity, BayK would equally modulate the function of pre- as well as postsynaptic Ca*_v_*1.x in a cumulative fashion that likely further boosts structural plasticity of the PSD. Future experiments will be required to test this hypothesis. The surprising additional finding that *oSparse* opto-STIM produces a strong modulatory phenotype reminiscent of functional inhibition and consequential homeostatic upscaling is puzzling, but might be explained by the rapid Ca^2+^ spike initiation process and resulting reduction in individual spike integrals that we observed in our patch clamp recordings: since the spike rates remained within the physiological range but lacked the slow initial depolarization phase, the attenuated Ca^2+^ influx might activate compensatory intracellular signaling pathways to increase release site efficiency. This hypothesis will also require extensive testing in future experiments.

## 4 Discussion

**Figure 9:**
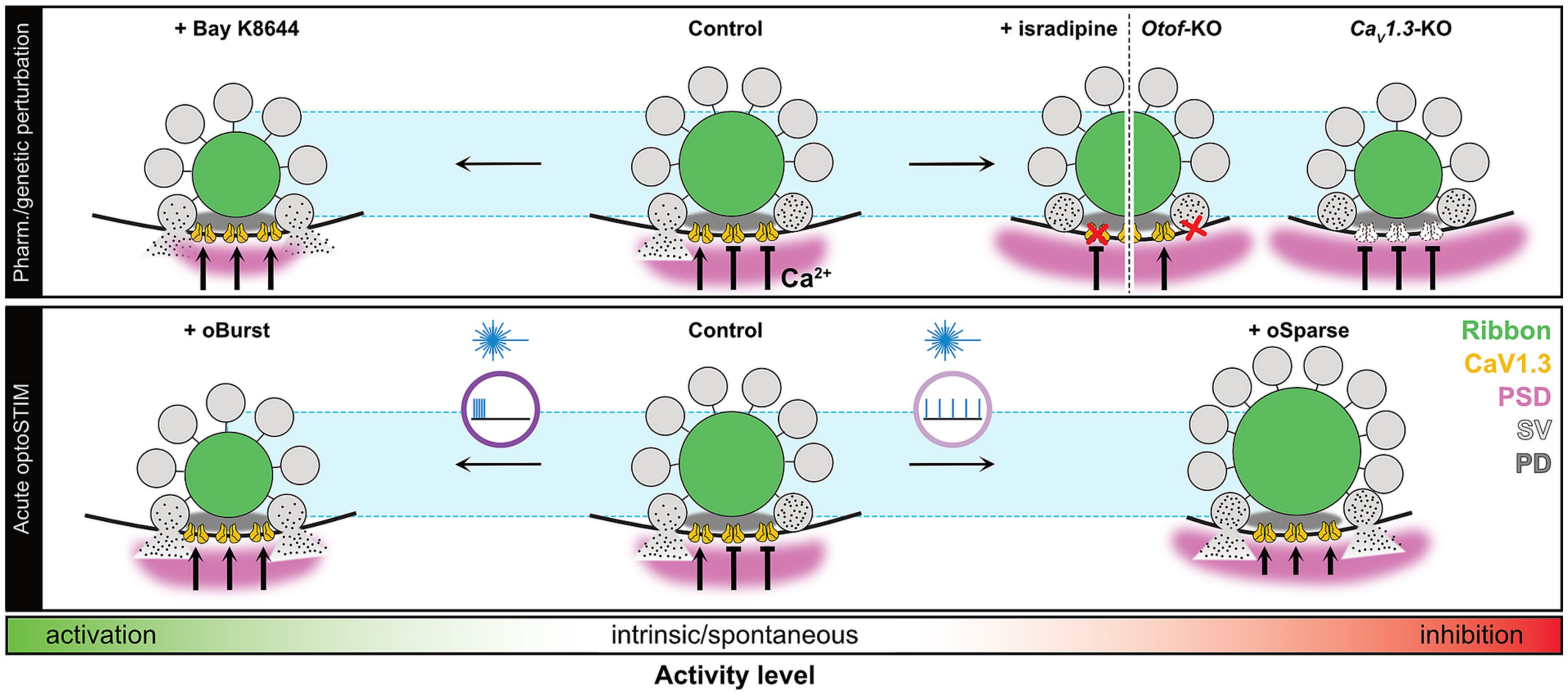
Summary of the observed structural changes of IHCs ribbon synapses following acute pharmacological, genetic, or optogenetic manipulation. Acute pharmacological inhibition of presynaptic Ca^2+^ influx using the L-type calcium channel inhibitor isradipine, or functional disruption of exocytosis due to absence of Otoferlin, resulted in unchanged ribbon size but an increase in PSD size. Genetic ablation of Ca*_v_*1.3 channels caused structural collapse of IHC ribbon size but also led to an increase in PSD size. Temporally-patterned optogenetic stimulation (*oSparse*) caused structural upscaling of both, ribbon and PSD size. In contrast, prolonged opening time of L-type calcium channels, induced by 1 h application of BayK led to a reduction in ribbon and PSD size. Notably, temporally-patterned optical stimulation in form of bursts (*oBurst*) similarly led to a reduction in ribbon size but failed to dramatically attenuate PSD size. Please note that we omitted possible postsynaptic Ca*_v_*1.*x* channels from this schematic drawing due to current lack of experimental evidence for their presence in the PSD. PD, presynaptic density; SV, synaptic vesicle.

In the present study, we investigated activity-dependent structural plasticity at cochlear IHC ribbon synapses by means of genetic, pharmacological, and optogenetic approaches. Specifically, using a combination of immunohistochemistry, functional imaging and patch-clamp electrophysiology, we tested the effects of pharmacological and genetics-based blanket inhibition or over-activation of synaptic function on IHC synaptic architecture and – in order to investigate differential temporal patterning as a key aspect of synapse size regulation – established a custom OSD for long-term and tightly-controllable in-incubator optogenetic stimulation. Using this model system, we show that positive as well as negative modulation of IHC presynaptic activity exerts rapidly-inducible opposing effects on ribbon synapse morphology that differentially affect both, the pre- as well as the postsynaptic architecture. Our findings are therefore compatible with activity-dependent homeostatic scaling of auditory ribbon synapses during maturation (Figure 9). Therefore, our study nicely complements previous work showing developmental structural plasticity of sensory ribbon synapses in zebrafish lateral line neuromasts hair cells (Sheets et al., 2012) and adds another layer of complexity to the pre-sensory maturational processes taking place in the cochlear periphery. By also including *Otof-*KOs IHCs into our analysis, our work additionally defocuses from pure Ca^2+^ influx modulation and thus contributes to a more holistic picture of maturational refinement processes. Next to the biological significance of our data, the herein detailed OSD description additionally provides a blueprint for an inexpensive and customizable light-based stimulation platform that may assist a wide range of future projects in unraveling activity-dependent cell biological processes in the auditory pathway and beyond.

### 4.1 Analysis of activity-dependent structural plasticity at mammalian IHC ribbon synapses – a challenging task

To date, most of our understanding of activity-dependent structural plasticity of ribbon synapses stems from work on illumination-dependent changes in ribbon morphology in retinal photoreceptors and pinealocytes of the pineal gland (Adly et al., 1999; Spiwoks-Becker et al., 2004, 2008; Dembla et al., 2020). In contrast to the highly accessible retina, where adequate photoreceptor activation is relatively easily achievable for rapid subsequent structure–function analyses, similar investigations of auditory IHCs are significantly more challenging: cochlear IHCs are deeply embedded in the temporal bone which makes *in situ* synapse monitoring a more difficult – yet somewhat solvable – task. However, due to the dissection procedure in which the sensory epithelium is excised from the boney cochlea and thus disconnected from the sound transduction pathway via the outer and middle ear, adequate auditory stimulation is impossible to achieve *in vitro.* To solve this conundrum, we resorted to a short-term organotypic organ of Corti culture model system, which has been essential in prior investigations of the origins of spontaneous activity in the developing auditory periphery as it offers easy experimental access – e.g., for pharmacological intervention – and tightly controllable maintenance conditions (Tritsch et al., 2010; Zhang-Hooks et al., 2016; Babola et al., 2020, 2021). To now add means to achieve temporally precise and customizable *in situ* activity modulation, we expanded this model system by employing an optogenetic mouse line for ChR2-expression in the IHC plasma membrane (Chakrabarti et al., 2022) and conceived an in-incubator OSD for tightly controllable light delivery in the millisecond range. Upon iterative optimization of OSD temperature management and power density as well as careful assessment of putative adverse effects – such as phototoxicity arising from the recurrent illumination – we are confident that the current OSD version produces safe, cell type specific and highly efficient light delivery to adequately cater the needs of long-term activity modulation of intact organotypically-cultured inner ear tissue.

### 4.2 Synaptic activity serves as a bi-directional driver of ribbon synapse structural maturation

As an initial application example for our new optogenetic model system, we assessed activity-dependent structural plasticity of IHC ribbon synapses. We found that developing ribbon synapses are plastic scaffolds that can rapidly modulate their size in a use-dependent manner: whereas (i) activity suppression or functional silencing by various acute (i.e., *oSparse* optoSTIM, L-type Ca*_v_*1.x inhibition via isradipine) or chronic approaches (i.e., *Ca_v_1.3*-KO, *Otof*-KO) led to AZ and/or PSD expansion, (ii) positive modulation of presynaptic activity (i.e., L-type Ca*_v_*1.x agonist BayK8644, *oBurst* optoSTIM) led to structural confinement of synaptic complexes. Hence, these findings are in line with previous research on zebrafish lateral line neuromast hair cell ribbon synapses (Sheets et al., 2012) and thus indicate mechanistic conservation throughout evolution. Moreover, our findings suggest that the PSD morphology is generally more susceptible to homeostatic regulation than the ribbon complex, which appears particularly robust to drastic inhibitory interventions such as acute pharmacological blockage of Ca*_v_*1.x Ca^2+^ influx. Importantly, our optoSTIM data indicate that functional silencing does not *per se* have no impact on ribbon synapse structure, but rather that ribbons appear more sensitive to subtle disruptions of temporal stimulus patterning and the associated finely-tuned presynaptic Ca^2+^ dynamics. In this context, it will be of major interest to test if the same rules apply for mature ribbon synapses – i.e., when the synaptic complex is fully established and developmental refinement has concluded – and delineate the roles of anterograde vs. retrograde signaling pathways during cochlear ribbon synapse maturation. Future studies will be required to investigate these processes in greater detail and our OSD presents an adequate tool to tackle such questions.

### 4.3 Activity patterns help determine adequate synapse maturation

One key finding of our study presents the fundamental importance of the temporal presentation of the activity, which is capable of differentially regulating synapse size in IHCs. Here, our data suggest that *oSparse* OSD stimulation synchronizes the IHC rhythmic firing and successfully ‘locks’ it to the optogenetically-imposed firing pattern, therefore effectively hijacking the intrinsic activity system. In fact, while regular firing events with compressed spike integrals – likely leading to an overall reduction in presynaptic Ca^2+^ influx – simulate a strong inhibitory-like upscaling response that involves the entire synaptic complex, transient bulk Ca^2+^ influx events during *oBurst* firing resemble activating pharmacological intervention that ultimately leads to synaptic downscaling. Future studies should test how more extreme firing pattern modulations – i.e., triggering either drastically higher, but also significantly lower firing rates – would affect this system and at what point the intrinsic spike generator capacity would be capable of overwriting such extrinsically imposed activity patterns.

### 4.4 Optogenetics as a useful tool to unravel synaptogenesis in the organ of Corti

Since its first implementation in modulating neuronal excitability and altering behavioral responses (Boyden et al., 2005; Nagel et al., 2005), optogenetics have proven an immensely useful tool for unraveling a plethora of cell biological processes. In our study, we used this method to investigate the effects of subtle activity modulation on ribbon synapse morphology in auditory IHCs and show that temporal stimulus patterning appears to play a fundamental role in synapse architecture. In fact, our findings indicate that the temporal presentation might even be of greater importance than the occurrence of spontaneous activity *per se.* Unlike pharmacological blanket inhibition/over-activation or global gene knock-out approaches – all of which eliminate or amplify select ion channel or other protein functions indiscriminately and regardless of their specific location – optogenetic activation adds instantaneous and exquisite target specificity as well as unmatched stimulus control, while operating in an otherwise largely unperturbed model system with intact Ca^2+^ channel performance, fully functional excitation-secretion coupling and maintained glutamate clearance from the synaptic cleft. Additionally, optogenetic stimulation has the added benefit of steering clear of developmental compensation mechanisms, which may complicate the interpretation of data obtained from excitability-related KO mouse strains. Finally, millisecond precise and solely presynaptic activity modulation will be invaluable in differentiating pre- from postsynaptic Ca*_v_*1.x contributions to ribbon synapse structural plasticity that could so far not be investigated with conventional tools.

### 4.5 Future perspectives

Exciting research directions using our OSD platform are manifold and, apart from the aforementioned antero- vs. retrograde regulation mechanisms, may also include investigations of the structural adaptations taking place in response to acute and/or persistent noise exposure. However, due to the customizability, high power and excellent temperature management also alternative experimental uses on other model systems – including neuronal cultures, acute brain slices or retinal preparations – could be envisioned. In fact, due to (i) the ease of technically adapting our OSD configuration for different LED/ChR2 requirements, (ii) the plethora of available opsins (Emiliani et al., 2022) and (iii) ever-increasing libraries of cell type specific promoters (for viral delivery) and *Cre recombinase* driver mouse lines, the possibilities seem somewhat limitless.

### 4.6 Limitations of the study

Next, to the obvious advantages of our model system, it is also important to highlight some of the shortcomings of our methodology: For example, our synapse size analyses are all endpoint measurements as it is currently not possible to continuously monitor the effects of persistent activity modulation throughout the stimulation period. This technical challenge could be resolved in the future by implementing optical stimulation for commercially available in-incubator microscopy solutions or custom-built setups to enable multi-color acquisition alongside optical stimulation; however, such applications will require means of fluorescently labeling intact ribbon synapses in living inner ear tissue (Voorn et al., 2025). The second major caveat to keep in mind when interpreting our results arises from the *in vitro* nature of our model system, which causes a major loss of efferent innervation and hence results in compromised regulatory input to afferent hair cell synapses. Future studies employing a recently reported *in vivo* microscopy approach may thus help in corroborating our findings (De Faveri et al., 2025). A final point to take into consideration is the fact that there are slight methodological differences between the light paths in our electrophysiological measurements and the OSD-based optoSTIM experiments: for patch clamping, light delivery occurred from hair bundle direction and was direct, whereas OSD-based illumination happened through the basilar membrane and had to pass a single layer of high precision cover-slip glass from the employed glass bottom dishes. This difference is owed to the technical requirements of the respective experiment and could therefore unfortunately not be homogenized between the approaches.

## Data Availability

The original contributions presented in the study are included in the article/Supplementary material, further inquiries can be directed to the corresponding author. All relevant source data are deposited on Mendeley Data for public access (Mendeley Data, doi: 10.17632/fkxph7grn5.1).
