# Supplementary material for: Patterned pre-sensory spontaneous activity drives the structural refinement of developing cochlear ribbon synapses
Source: Front Synaptic Neurosci. 2026 Mar 23;18:1730181. doi: 10.3389/fnsyn.2026.1730181 (PMC13050871; doi:10.3389/fnsyn.2026.1730181)
Supplement: Supplementary file 1 [file Data_Sheet_1.pdf]

Supplementary Material

Patterned pre-sensory spontaneous activity drives the structural refinement of developing cochlear ribbon synapses

Authors: Victoria C. Halim [1,2], Lukas Hallbrucker [1], Jan F. Ahrend [1,3], Cristian Setz [2,4,5], Roos A. Voorn [1,2,5], Samira Franke [1,2], Vanessa Konrad [1], Alina Seiler [2], Tina Pangršič [2,4,5], Stefan Roesler [6] and Christian Vogl [1,2,3,5*]

Affiliations:

[1] Auditory Neuroscience Group, Institute of Physiology, Medical University Innsbruck, Innsbruck, Austria

[2] Institute for Auditory Neuroscience and InnerEarLab, University Medical Center Göttingen, Göttingen, Germany

[3] *Ca_VX – Calcium channels in excitable cells* PhD Program, Medical University Innsbruck, Innsbruck, Austria

[4] Department of Otolaryngology, Head and Neck Surgery, University Medical Center Göttingen, Germany

[5] Collaborative Research Centre 889 '*Cellular Mechanisms of Sensory Processing*', Goettingen, Germany

[6] Intas Science Imaging Instruments GmbH, Göttingen, Germany

*Correspondence should be addressed to: christian.vogl@i-med.ac.at

1 Supplementary Figures and Tables

1.1 Supplementary Figures

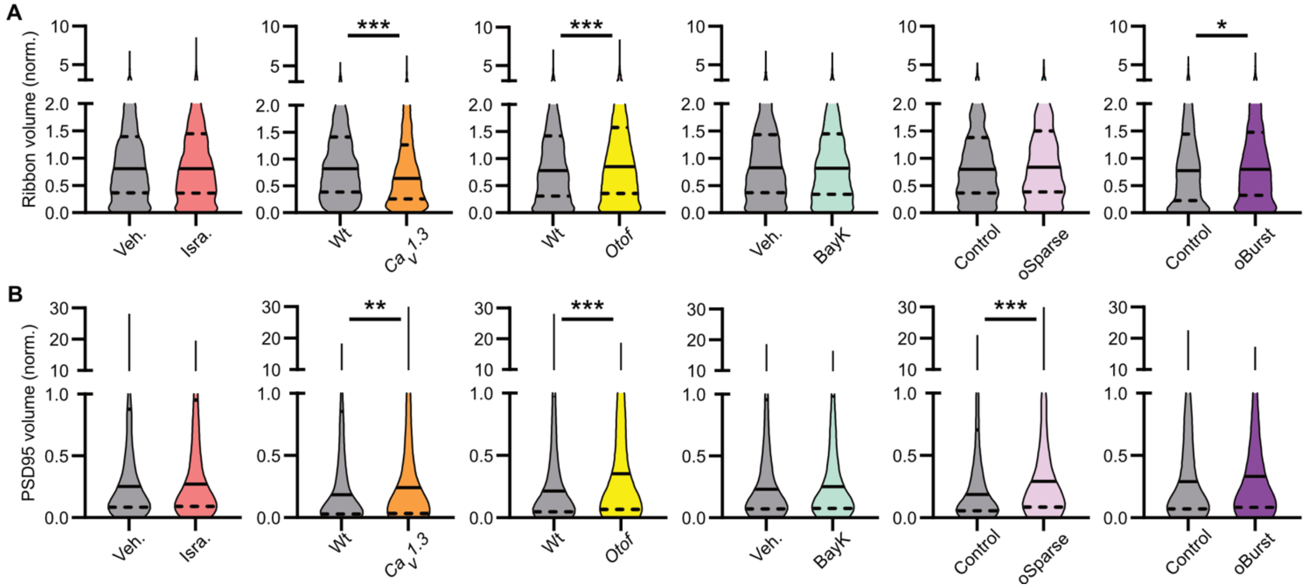

Supplementary Figure S1. Summary of synaptic volume modulation upon acute pharmacological, genetic or optogenetic activity modulation.

(A-B) Violin plots comparing normalized volumes of reconstructed (A) presynaptic ribbons and (B) postsynaptic PSD95 patches in P5DIV1 IHCs between all investigated test conditions (isradipine, *Ca$_V$1.3*-KO, *Otof*-KO, BayK, oSparse, oBurst) and their respective controls. N-numbers for each condition are identical with the ones mentioned in the corresponding main Figures. Medians and interquartile ranges are indicated by solid and dashed lines inside the violin plots. Mann-Whitney U-test: *P < 0.05, **P < 0.01, ***P < 0.001.

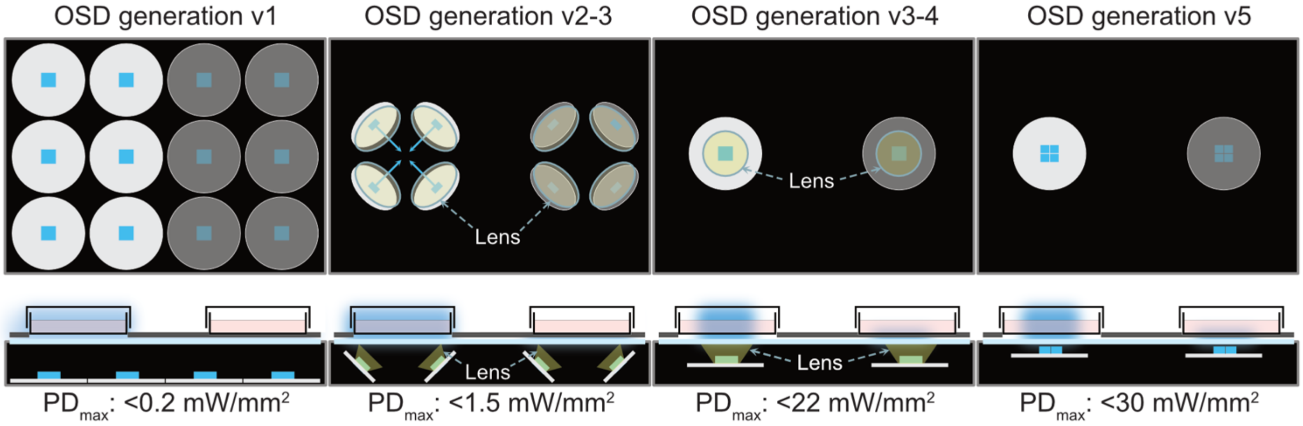

Supplementary Figure S2. Design iterations of the optical stimulation device.

Schematic drawings of the trialed design concepts for the OSD illustrating different LED positions and configurations in top view (upper panels) or side view (lower panels). In v3-4 the use of focus lenses was additionally tested. Indicated below the drawings are the maximally achieved power density (PD) values per OSD generation. All experiments in the main manuscript have been performed with OSD v5.

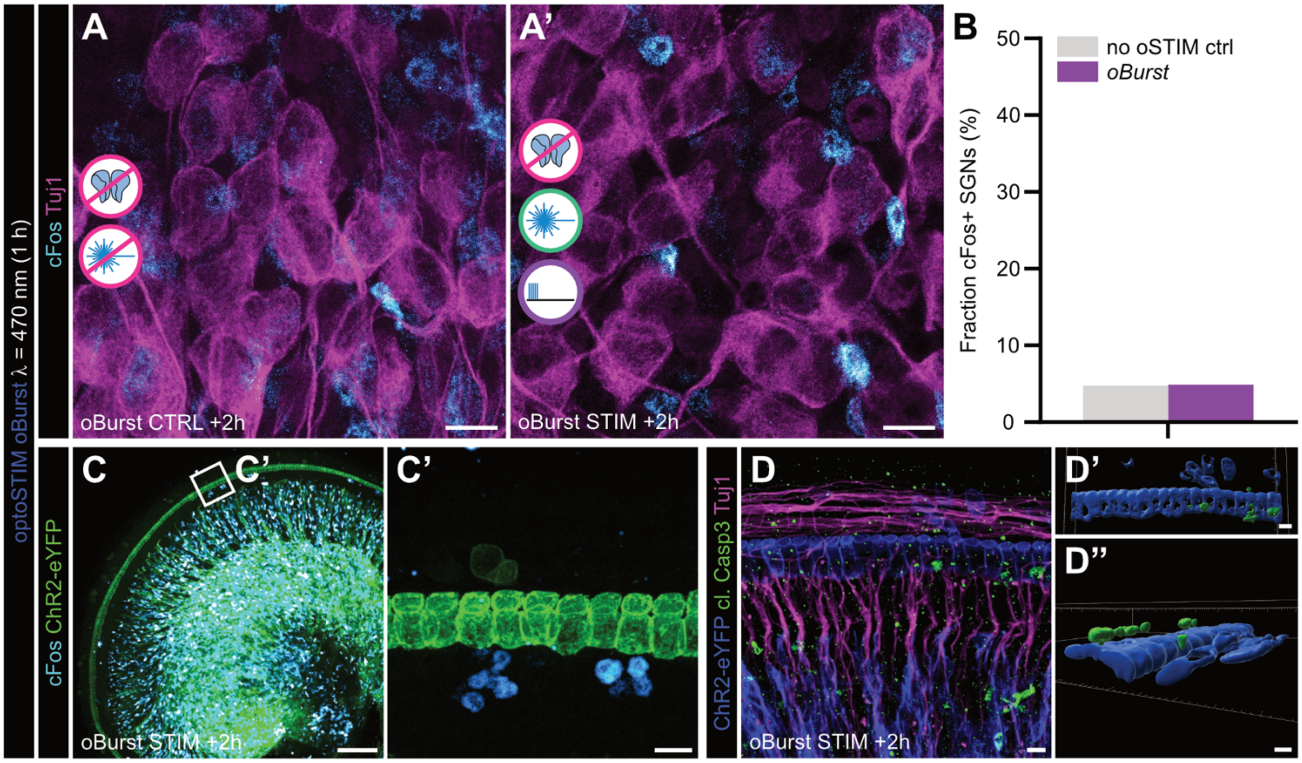

Supplementary Figure S3. Light stimulation in the absence of ChR2 does not trigger cFos expression.

(**A-A'**) cFos expression in SGNs after 1 h optical stimulation (optoSTIM) of Ai32-Vglut3^{Cre-} IHCs – which lacked ChR2 expression – and evaluated 2h after stimulus onset. Representative confocal images of the spiral ganglion of P5DIV1 organs of Corti 2 h after (A) no optical stimulation or (A') oBurst stimulation ($N_{animals}$ = 5, n_{Corti} = 5 for both control and oBurst), SGNs were stained for cFos (cyan) and Tuj1 (magenta). Scale bars: 10 µm.

(B) Quantification of cFos+ SGNs shows comparable results between *oBurst* CTRL and *oBurst* STIM of Ai32-Vglut3^{Cre-} organs of Corti ($N_{animals}$ = 5 for both conditions, n_{SGN} = 187 and 184 for oBurst CTRL and oBurst STIM respectively).

(C-C') Representative confocal maximum z-projection (C) overview and (C') high magnification image of hair cells in an Ai32-Vglut3^{Cre+} organ of Corti after *oBurs*t STIM illustrating the absence of cFos expression in IHCs. Note that few supporting cells are positive for cFos. Organs of Corti were stained for eYFP (green) and cFos (cyan). Scale bars: 100 µm and 10 µm.

(D-D'') Representative confocal maximum z-projection overview image, (D') top view (D'') sideview of surface reconstructed IHCs of Ai32-Vglut3^{Cre+} organs of Corti after *oBurst* stimulation representing absence of apoptosis in IHCs. Organs of Corti were stained for eYFP (blue), Tuj1 (magenta), and cleaved Caspase-3 (green) as a marker for cell apoptosis. IHCs and cleaved Caspase-3 puncta were reconstructed using Imaris 11.0.0. Scale bars: 10 µm.

1.2 Supplementary Tables

Supplementary Table S1: Resources table

REAGENT or RESOURCE	SOURCE	IDENTIFIER
**Biological sample**		
Organ of Corti explant cultures	C57BL/6J mice (Jackson Laboratory)	JAX ID: #000664
	C57BL/6NJ (The Jackson Laboratory)	JAX ID: #005304
	Otof-KO mice	(Reisinger et al., 2011)
	$Ca_v 1.3$-KO mice	(Platzer et al., 2000)
	Ai32-Vglut3-Cre knock-in mice	(Chakrabarti et al., 2022)
	B6.Cg-*Snap25*$^{tm3.1Hze}$/J (Snap25-2A-GCaMP6s-D, The Jackson Laboratory)	JAX ID: #025111
**Antibodies**		
anti-Post Synaptic Density Protein 95, clone 7E3-1B8	Millipore	MAB1598
anti-Tubulin β3 (TUBB3) clone TuJ1	BioLegend	801202
anti-Cleaved Caspase-3 (Asp175)	Cell Signaling Technology	9661S
Anti-Myosin7a	DHSB	MYO7A 138-1
anti-Parvalbumin	Synaptic Systems	195 004
anti-Ribeye A-domain	Synaptic Systems	192 103
anti-c-Fos	Synaptic Systems	226 008
anti-CtBP2	BD Biosciences	612044
anti-Ca_v1.3	Alomone (KO-verified)	ACC-005
anti-Homer1	Synaptic Systems	160 006
Anti-GFP FluoTagX4 Atto488	NanoTag	N0304- At488-L
Anti-mouse FluoTagX2 IgG1 635p	NanoTag	N2002- Ab635p
Anti-mouse FluoTagX2 IgG2a/b 565	NanoTag	N2702-At565s
Goat anti-mouse IgG1 594	Invitrogen	A21125
Anti-mouse FluoTagX2 IgG2a/b 635p	NanoTag	N2702- Ab635p-S
Anti-rabbit FluoTagX4 580	NanoTag	N2404- Ab580-S
Goat anti-chicken Alexa-488	ThermoFisher Scientific	A11039
Goat anti-guinea pig 488	ThermoFisher Scientific	A-11073
Goat anti-rabbit 488	Invitrogen	A11008
Goat anti-mouse Abberior STAR-580	Abberior GmbH	2-0002-005-1
Goat anti-rabbit 647	Abcam	ab150079
Goat anti-rabbit Abberior STAR-635p	Abberior GmbH	2-0012-007-2
TO-PRO™-3 Iodide 642/661	ThermoFisher Scientific	T3605
**Chemicals and reagents**		
Ampicillin	Sigma	A0166
Amphotericin B	Merck	171375
B27	Life Technologies	17504044
BayK8644	Tocris	1544
Cell-Tak™	Corning	354240
Dimethyl sulfoxide Hybri-Max™	Sigma	D2650
Fungizone	Life Technologies	15290-026
GlutaMAX™-1 (100X)	Gibco	35050-061
HBSS (1X)	Gibco	14025-092

**Chemicals and reagents (continued)**		
HEPES	Gibco	15630-049
Isradipine	Sigma	I6658
KCl	Roth	6781
$MgCl_2$	Sigma	63020
Neurobasal-A medium (1X)	Gibco	12349-015
Penicillin G	Sigma	P3032
ProLong™ Glass Antifade Mountant	Life Technologies	P36984
**Software**		
GraphPad Prism	GraphPad Software	https://www.graphpad.com/
ImageJ/FIJI	Schneider et al.,2012	https://imagej.nih.gov/ij
Imaris (x64 9.6.1)	Oxford Instruments	imaris.oxinst.com
Imaris (x64 10.2.0)	Oxford Instruments	imaris.oxinst.com
OSD driver software: Vogl-OSD (1.0.0.1)	Intas Science Imaging Instruments	www.intas.de
**OSD Materials**		
Housing	Reichelt Elektronik	KL1505.510 500 x 200 x 122
Switch	Reichelt Elektronik	WIPPE 1805.7110
Power Supply	MeanWell	RS-50-24
LED driver	MeanWell	LDD-350H
	MeanWell	LDD-700H
	MeanWell	LDD1000H
Connector sockets	Bulgin	PX0412/02S
PCB, Imager-Interface Rev.CA with Atmel	Intas Science Imaging Instruments	ATMEGA64A microcontroller
Connector plugs	Bulgin	PX0410/02P/5560
Cable	BELI-BECO	YL6220
Thermal sensor (temperature and humidity USB sensor probe)	Thorlabs	TSP01
Illumination unit housing	Bopla Aluplan	AP 51015
Heatsink bottom	Fischer Elektronik	SK 92 75 SA
Illumination unit cover	Plexiglas XT white	WN670 GT, transmittance 78%
Illumination unit mount	Fischer Elektronik	10020382 SK 46 20
Series Connected Blue LEDs	LUXEON Z	
Z5 20mm Base	Saber	SZ-05-H3 470 nm 152 lm
